# The blood metabolome of brain health in midlife and influences of genes, microbiome and exposome

**DOI:** 10.1038/s43587-026-01149-4

**Published:** 2026-06-24

**Authors:** Shahzad Ahmad, Tong Wu, Matthias Arnold, Thomas Hankemeier, Mohsen Ghanbari, Gennady Roshchupkin, André G. Uitterlinden, Kamil Borkowski, Julia Neitzel, Robert Kraaij, The Alzheimer’s Disease Metabolomics Consortium, Matthias Arnold, Thomas Hankemeier, Cornelia M. van Duijn, M. Arfan Ikram, Rima Kaddurah-Daouk, Gabi Kastenmüller, Cornelia M. van Duijn, M. Arfan Ikram, Rima Kaddurah-Daouk, Gabi Kastenmüller

**Affiliations:** 1 Department of Epidemiology, Erasmus MC, University Medical Center, Rotterdam, The Netherlands; 2 Division of Systems Biomedicine and Pharmacology, Leiden Academic Center for Drug Research, Leiden University, Leiden, The Netherlands; 3 Oxford-GSK Institute of Molecular and Computational Medicine (IMCM), Centre for Human Genetics, Nuffield Department of Medicine, University of Oxford, Oxford, UK; 4 Sidra Medicine, Doha, Qatar; 5 Institute of Computational Biology, Helmholtz Zentrum München, German Research Center for Environmental Health, Neuherberg, Germany; 6 Department of Psychiatry and Behavioral Sciences, Duke University, Durham, NC USA; 7 Department of Internal Medicine, Erasmus MC, University Medical Center, Rotterdam, The Netherlands; 8 West Coast Metabolomics Center, Genome Center, University of California, Davis, Davis, CA USA; 9 Department of Radiology and Nuclear Medicine, Erasmus MC, University Medical Center, Rotterdam, The Netherlands; 10 Nuffield Department of Population Health, University of Oxford, Oxford, UK; 11 Duke Institute of Brain Sciences, Duke University, Durham, NC USA; 12 Department of Medicine, Duke University, Durham, NC USA

**Keywords:** Systems analysis, Metabolomics, Data integration, Alzheimer's disease, Ageing

## Abstract

Metabolic alterations are increasingly implicated in neurological disorders, including Alzheimer’s disease (AD), highlighting the relevance of the peripheral metabolome, shaped by genetic and environmental exposures, for brain health. We examined the relation of 991 blood metabolites with cognition and magnetic resonance imaging (MRI) measures cross-sectionally in 1,082 dementia-free middle-aged participants of the population-based Rotterdam Study and quantified contributions of genetic variation, lifestyle, comorbidities, medication and gut microbiota to metabolite variance. Cognition-associated metabolites were replicated in two independent cohorts of older adults and tested for associations with incident AD longitudinally in one cohort. Twenty-two metabolites were associated with MRI measures. Fourteen metabolites showed replicated associations with cognition, with ergothioneine exhibiting the largest effect. The metabolite signature of cognition mirrored that of incident AD. Lifestyle, clinical variables and medication were the strongest determinants of cognition-associated and MRI-associated metabolites, explaining up to 28.6% of their variance. Antacid use was associated with worse cognition and lower ergothioneine levels, which mediated 31.5% of the negative medication effect, suggesting implications for AD prevention.

## Abstract (web summary)

Metabolic alterations have been associated with age-related neurodegenerative diseases. Here the authors examine the peripheral human metabolome and show that metabolite profiles connect midlife exposures to brain health. The research links circulating metabolites influenced by lifestyle and medication to cognition, MRI markers and Alzheimer’s disease risk.

## Main

Growing evidence implicates alterations of the peripheral metabolism in brain-related diseases, including AD^1–3^. Multiple studies have linked the blood levels of various metabolites, including amino acids (for example, valine), lipids (including acylcarnitines, phosphatidylcholines, sphingomyelins and bile acids) and lipoproteins, with AD-related phenotypes such as cognition and brain imaging phenotypes or the conversion from a cognitively normal state to AD^4–15^.

Highlighting the long presymptomatic phase of AD, structural and functional changes in various brain regions and alterations in neuropsychological markers emerge many years before disease manifestation^16–19^. In particular, brain atrophy (cortical thinning and reduction in hippocampal volume) and the presence of white matter hyperintensities as detected through MRI serve as distinctive neurodegenerative and vascular markers associated with AD^20–22^. Studying early metabolic changes linked with these features of AD, before the onset of any symptoms, might reveal metabolites that are important in the disease etiology.

Blood metabolomes reflect metabolic states as resulting from the interplay among genetics, gut microbiome and exposome^23,24^. In recent studies, the gut microbiome and the exposome, which includes lifestyle factors, medication use and further environmental exposures, emerged as important contributing factors to influence metabolite levels in addition to genetics^25–28^. For example, by building a reference map of potential determinants of the human serum metabolome in an Israeli cohort^25^, diet and gut microbiome were shown to play a crucial role in defining the metabolic repertoire within the systemic circulation.

As the gut microbiome and many factors of the exposome are modifiable in nature, their management provides an opportunity to stabilize metabolism and counteract disease-related metabolic alterations. However, to define targeted interventions and prevention strategies, a profound understanding is required of (1) the metabolites associated with early alterations in brain structure and cognitive function related to AD and (2) the extent to which these metabolite levels are influenced (and, thus, potentially modifiable) by the gut microbiome and the exposome in presymptomatic individuals. Despite increasing knowledge on metabolites associated with cognition, MRI markers and AD diagnosis in older adults, the implications of metabolites for brain health in midlife, their specific interplay with risk factors and potential role as mediators of risk are not well understood.

In the present study, we aimed at filling this research gap by systematically combining the identification of early metabolic markers of brain health with the analysis of their potential determinants, including various metabolism-related and AD-related risk factors. We hypothesize that this integrated analysis can pinpoint metabolites that may partially convey the effects of exposomal and lifestyle factors on brain function with potential consequences for prevention. Specifically, we first investigated the cross-sectional association of circulating metabolites with general cognition and MRI markers in midlife within a dementia-free sample of the population-based Rotterdam Study to identify potential biomarkers of brain health. The resulting cognition-associated metabolites were replicated cross-sectionally in two independent cohorts of older individuals. To ensure the relevance of these metabolites for AD, they were further tested for their relationship with incident AD diagnosis during follow-up in one of the replication cohorts in a prospective design. In a second step, we evaluated the contribution of genetic variation, gut microbiome, various lifestyle factors, medication use and clinical features to define levels of identified metabolites in circulation in the discovery cohort and investigated the interplay of these factors in detail for selected examples, exploring their potential as targets for intervention and prevention.

## Results

For this study, the levels of 1,387 metabolites were determined in plasma samples from 1,082 participants of the RSIII-2 cohort (that is, the third cohort at second follow-up) of the Rotterdam Study using a nontargeted metabolomics approach (Metabolon HD4). To elucidate the connection of the peripheral metabolome with brain health and function, we first investigated the cross-sectional association of general cognition and MRI markers with the levels of 991 frequent metabolites in the 1,068 RSIII-2 participants (after data preprocessing) (Methods and Supplementary Fig. 1). For cognition, we replicated our findings in 874 participants of the older RSI-4 cohort, an independent sample (that is, separate survey, no overlap with discovery cohort) of the Rotterdam Study, for which general cognition (*g*factor) and metabolomics data from the same platform were available at the same timepoint. The 874 RSI-4 participants used for the replication analysis did not have any diagnosis of dementia (or stroke) at this time or during later clinical follow-ups. As a second replication analysis, we tested the association of the cognition-associated metabolites with three cognitive scores in a US-based cohort, in which data from the same metabolomic platform were available for 10 of the 14 metabolites.

Furthermore, to ensure the relevance of our findings for AD, we also compared the cross-sectional metabolomic signature of cognition from the discovery cohort with the signature of incidence of AD prospectively in RSI-4. For this analysis, we included metabolomics data from 355 additional participants who were dementia free at the time of metabolomics measurement but were diagnosed with AD during longitudinal clinical follow-ups, yielding a total of 1,229 RSI-4 participants available for Cox regression. Characteristics of participants from the Rotterdam Study discovery and replication cohorts are summarized in Table 1.

**Table 1:** Population characteristics

Study characteristics	Rotterdam Study III	Rotterdam Study I	Rotterdam Study I
	(*n* = 1068)	(*n* = 874)	(*n* = 355)
AD status at follow-up	No follow-up	No AD^a^	Incident AD^b^
Age in years, mean (s.d.)	62.54 (5.91)	76.25 (4.78)	78.13 (6.17)
Sex, female (%)	598 (56)	506 (57.89)	234 (65.92)
BMI, kg m^−2^, mean (s.d.)	27.35 (4.30)	27.54 (4.23)	27.00 (4.03)
Smoking status (%)			
Never	350 (32.56)	250 (28.60)	124 (35.73)
Former	574 (54.42)	531 (60.75)	193 (55.62)
Current	140 (13.02)	93 (10.64)	30 (8.65)
Educational attainment (%)			
Primary education	422 (39.62)	519 (59.93)	230 (65.16)
Further education	308 (28.92)	257 (29.67)	92 (26.06)
Higher education	335 (31.45)	90 (10.39)	31 (8.78)
Hypertension (%)	497 (46.66)	506 (57.89)	205 (57.91)
Total cholesterol, mmol l^−1^, mean (s.d.)	5.578 (1.12)	5.56 (0.95)	5.682 (0.95)
HDL cholesterol, mmol l^−1^, mean (s.d.)	1.507 (0.45)	1.44 (0.39)	1.492 (0.40)
Use of lipid-lowering drugs (%)	286 (12)	185 (21.16)	74 (20.85)
Diabetes (%)	65 (6.09)	62 (7.11)	29 (8.17)

^a^Participants did not have any diagnosis of dementia or stroke at time of metabolomics measurement or during follow-up.

^b^Participants were dementia free at time of metabolomics measurement but were diagnosed with AD during follow-up.

To explore the potential of modifying the cognition-associated and MRI marker-associated metabolites through interventions, we assessed how much metabolite levels were influenced by modifiable versus unmodifiable features as a second step. For this purpose, we estimated the portion of metabolite variance that is explained by the variation in the participants’ gut microbiome, lifestyle, clinical factors, medication and genetics for all metabolites and additionally investigated the pairwise cross-sectional association between each factor and metabolite in the discovery cohort. Details of the performed analyses are provided in the Methods section. A general overview over the steps is given in Supplementary Fig. 2.

### General cognition and brain MRI markers are associated with distinct blood metabolites

**Fig. 1:**
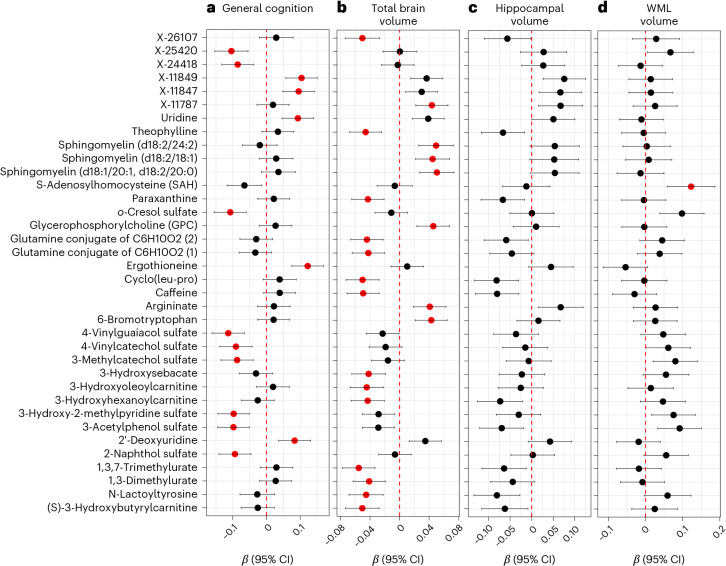
Associations of plasma metabolites with cognition and brain imaging traits. Forest plots show the association of metabolites with general cognition (**a**), total brain volume (**b**), hippocampal volume (**c**) and WML volume (**d**). Points (center) represent effect estimates (*β*) from linear regression models, and horizontal bars represent 95% confidence intervals (CI). Red points indicate metabolites that are significant after FDR correction (FDR < 0.05). Note that N-lactoyltyrosine is the updated annotation of a metabolite that was annotated as 1-carboxyethyltyrosine in the original dataset. This correction was provided by Metabolon. Source data

**Extended Data Fig. 1:**
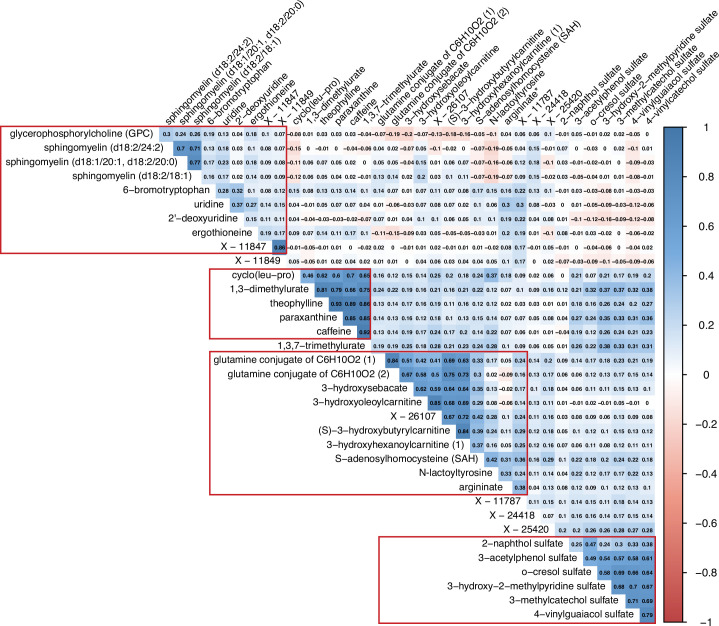
Correlation matrix of metabolites associated with general cognition and brain imaging markers. Heatmap showing pairwise Spearman correlation coefficients between the 36 brain health-associated (FDR < 0.05) metabolites. Colors represent the strength and direction of correlations, ranging from -1 (red) to 1 (blue), as indicated by the color scale. Values displayed in each cell correspond to the Spearman correlation coefficients between metabolite pairs. Red boxes around metabolite names highlight clusters of highly correlated metabolites. Note that N-lactoyltyrosine is the updated annotation of a metabolite that was annotated as 1-carboxyethyltyrosine in the original data set. This correction was provided by Metabolon. Source data

Analyzing the relationship of 991 metabolites with general cognition cross-sectionally, we observed significant associations (false discovery rate (FDR) < 0.05) of 14 metabolites with cognition while adjusting for age, sex, body mass index (BMI) and lipid-lowering medication use (model 1) (Fig. 1, Table 2 and Supplementary Table 1). Higher levels of ergothioneine (adjusted mean difference = 0.122, *P* = 4.65 × 10^−7^), uridine (0.093, *P* = 1.0 × 10^−4^), 2-deoxyuridine (0.083, *P* = 5.48 × 10^−4^) and two chemically uncharacterized metabolites (X-11849 and X-11847) were associated with better cognition. Moreover, lower levels of seven sulfated xenobiotic metabolites (4-vinylguaiacol sulfate, *o*-cresol sulfate, 3-acetylphenol sulfate, 3-hydroxy-2-methylpyridine sulfate, 2-naphthol sulfate, 4-vinylcatechol sulfate and 3-methylcatechol sulfate) and two uncharacterized metabolites (X-25420 and X-24418) showed association with better general cognition. These metabolites fall into three main sets of correlated metabolites (Extended Data Fig. 1), namely (1) sulfated xenobiotics (0.47 < *r* < 0.79), (2) the nucleotides 2′-deoxyuridine and uridine (*r* = 0.37) and ergothioneine (*r* = 0.27) and (3) the uncharacterized metabolites X-11849 and X-11847 (*r* = 0.86).

**Table 2:** Metabolites associated with general cognition

Metabolite	Discovery cohort (RSIII-2)				Replication cohort (RSI-4)		
	*β*	s.e.	*P* value	FDR	*β*	s.e.	*P* value
Ergothioneine	0.122	0.024	4.65 × 10^−^^7^	4.61 × 10^−4^	0.073	0.028	9.60 × 10^−3^
4-Vinylguaiacol sulfate	−0.114	0.024	3.85 × 10^−6^	1.91 × 10^−3^	-0.137	0.029	1.75 × 10^−6^
*o*-Cresol sulfate	−0.107	0.024	1.00 × 10^−5^	3.32 × 10^−3^	-0.148	0.028	1.83 × 10^−7^
X-11849	0.104	0.024	1.41 × 10^−5^	3.48 × 10^−3^	0.016	0.028	5.62 × 10^−1^
3-Acetylphenol sulfate	−0.098	0.024	4.41 × 10^−5^	7.28 × 10^−3^	-0.079	0.028	5.02 × 10^−3^
X-25420	−0.104	0.025	3.73 × 10^−5^	7.28 × 10^−3^	-0.059	0.030	5.04 × 10^−2^
3-Hydroxy-2-methyl-pyridine sulfate	−0.097	0.024	5.22 × 10^−5^	7.40 × 10^−3^	-0.090	0.028	1.40 × 10^−3^
X-11847^a^	0.095	0.024	7.07 × 10^−5^	8.75 × 10^−3^	0.002	0.028	9.31 × 10^−1^
Uridine	0.093	0.024	1.00 × 10^−4^	1.10 × 10^−2^	0.020	0.028	4.77 × 10^−1^
2-Naphthol sulfate	−0.094	0.024	1.11 × 10^−4^	1.10 × 10^−2^	-0.103	0.028	2.46 × 10^−4^
4-Vinylcatechol sulfate	−0.091	0.024	2.16 × 10^−4^	1.95 × 10^−2^	-0.103	0.028	2.81 × 10^−4^
3-Methylcatechol sulfate^a^	−0.087	0.024	3.53 × 10^−4^	2.92 × 10^−2^	-0.091	0.028	1.37 × 10^−3^
X-24418	−0.086	0.024	4.08 × 10^−4^	3.11 × 10^−2^	-0.052	0.028	6.66 × 10^−2^
2′-Deoxyuridine	0.083	0.024	5.48 × 10^−4^	3.88 × 10^−2^	0.061	0.028	2.79 × 10^−2^

Results from model 1 (linear regression; covariates: age, sex, BMI and lipid-lowering medication); ‘^a^’ indicates metabolites not significant in model 2 (linear regression; covariates: model 1 + education). Multiple testing correction was performed controlling for FDR < 0.05 in the discovery analysis.

After adjustment for education (model 2) (Supplementary Table 1), the associations for 12 of the 14 metabolites remained significant (FDR < 0.05). After further adjustment for smoking, diabetes and hypertension (model 3; Supplementary Table 1), all 14 metabolites associated with cognition in model 1 still showed evidence of association (*P* < 0.05) with cognition but did not reach FDR*-*adjusted significance.

For the MRI markers, we observed significant association between 21 metabolites with total brain volume and one metabolite (S-adenosylhomocysteine (adjusted mean difference = 0.123, *P* = 1.73 × 10^−4^)) with white matter lesion (WML) volume, whereas none was associated with hippocampal volume (Fig. 1 and Supplementary Table 2). Among the significant metabolites, higher levels of three sphingomyelins, glycerophosphorylcholine (GPC), 6-bromotryptophan, argininate and X-11787 were associated with higher brain volume. By contrast, lower levels of six metabolites related to the caffeine degradation pathway, N-lactoyltyrosine and seven intercorrelated metabolites (0.41 < *r* < 0.85; Extended Data Fig. 1), including three hydroxylated acylcarnitines and a hydroxylated dicarboxylic fatty acid, showed associations with higher brain volume. N-Lactoyltyrosine correlated with S-adenosylhomocysteine (SAH), which was associated with WML (*r* = 0.42), and 6-bromotryptophan moderately correlated (*r* = 0.32) with 2′-deoxyuridine associated with cognition.

All 22 metabolites were associated with MRI phenotypes at *P* < 0.05 when models were additionally adjusted for smoking, diabetes and hypertension (model 2; Supplementary Table 2). After correcting for multiple testing, seven of the 22 metabolites remained significant at FDR < 0.05. In sensitivity analyses, excluding participants with more than a 1-year interval between MRI and blood collection for metabolomics (leaving *n* = 688 participants), all 22 metabolites remained associated at *P* < 0.05. Of the 21 metabolites associated with total brain volume, 12 were also significant after adjustment for testing 991 metabolites (FDR < 0.05). Also, the association of SAH with WML remained significant in the sensitivity analysis at FDR < 0.05 (Supplementary Table 2).

Although none of the 991 metabolites showed a significant association with hippocampal volume, 17.5% of its variance could be explained by an elastic net regularization model trained on the full list of metabolites. Analogous models for total brain volume, WML volume and general cognition explained 32.69%, 1.98% and 10% of their variance, respectively (Supplementary Table 3).

### Metabolite signatures of cognition and MRI markers are concordant

**Extended Data Fig. 2:**
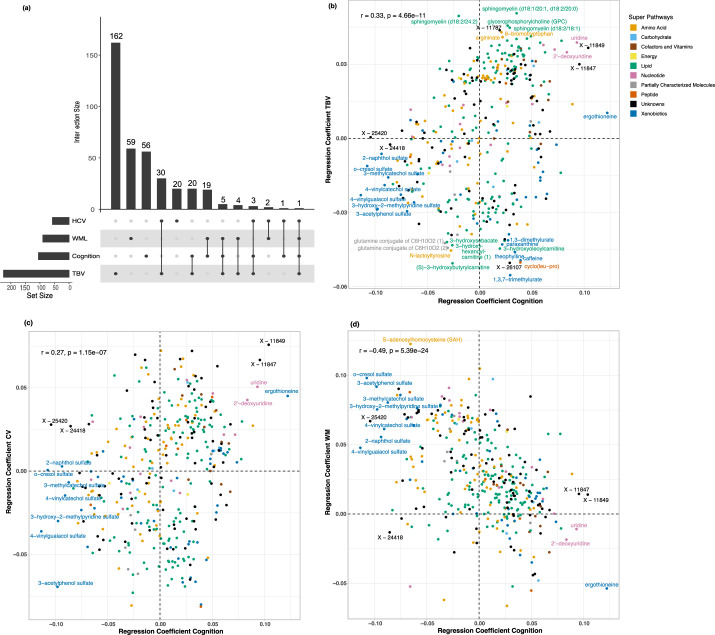
Concordance of metabolite signatures between cognition and brain imaging markers. (**a**) UpSet plot showing the overlap of metabolites associated with general cognition (*g*factor), total brain volume (TBV), hippocampal volume (HCV) and white matter lesion (WML) volume at nominal significance *P* < 0.05 (two sided) in regression analyses. Scatter plot showing the regression coefficients of the metabolites associated with TBV (**b**), HCV (**c**) and WML volume (**d**) versus the regression coefficients of the metabolites with general cognition (including all metabolites with *P* < 0.05 in the association to the brain imaging marker or to cognition). The color indicates the class of metabolites and the Pearson’s correlation coefficient () between regression coefficients is shown in each panel with the corresponding two-sided *P* value. Source data

Although the sets of cognition-associated and MRI-associated metabolites did not overlap, their effect patterns (Fig. 1) suggested similarities. We, therefore, tested the overall concordance of metabolite signatures across these related phenotypes by assessing the correlation of the regression coefficients for cognition and MRI markers, including all metabolites that showed nominal association (*P* < 0.05) to one of the compared phenotypes. Regression coefficients for the metabolite associations of cognition significantly correlated with the coefficients from the association with total brain volume (Extended Data Fig. 2b; *r* = 0.33, *P* = 4.66 × 10^−11^), with hippocampal volume (Extended Data Fig. 2c; *r* = 0.27, *P* = 1.15 × 10^−7^) and with WML volume (Extended Data Fig. 2d; *r* = −0.49, *P* = 5.39 × 10^−24^).

Out of 105 metabolites that nominally associated (*P* < 0.05) with cognition, 49 also showed nominal association with at least one of the three MRI phenotypes (Extended Data Fig. 2a). 3-Acetylphenol sulfate was the only metabolite that showed significant association with general cognition (adjusted mean difference = −0.098, *P* = 4.41 × 10^−5^) and nominal association with all three MRI phenotypes (total brain volume (−0.029, *P* = 9.59 × 10^−3^), hippocampal volume (−0.069, *P* = 7.89 × 10^−^^3^) and WML volume (0.092, *P* = 2.24 × 10^−^^3^)). Moreover, of the 14 metabolites significantly associated with cognition, concordant effects were observed for ergothioneine between cognition, hippocampal volume and WML volume and for uridine, 2′-deoxyurdine, X-11847 and X-11849 between cognition, hippocampal volume and total brain volume but not WML volume. Conversely, the uncharacterized metabolites X-24418 and X-25420 did not show any effects in the association with MRI phenotypes.

### Metabolite signature of cognition replicates in two cohorts and overlaps with AD signature

Among the 14 metabolites associated with general cognition in RSIII-2 (FDR < 0.05), nine showed significant association (*P* < 0.05) with concordant effect directions (Table 2) in the cross-sectional replication analysis, which was based on 847 participants from the RSI-4 cohort recruited independently from the same region in The Netherlands 15 years before RSIII recruitment.

For further replication, we used data from seven Alzheimer’s Disease Research Centers (ADRC) across the United States collected for 512 patients as part of the Alzheimer Gut Microbiome Project (AGMP). We tested the cross-sectional associations of three cognitive scores (Craft Story Delayed Recall (CRAFTDRE), Uniform Data Set Benson Figure Total Delayed (UDSBENTD) and education-adjusted total National Alzheimerʼs Coordinating Center Montreal Cognitive Assessment (NACCMOCA)) with 10 of the 14 cognition-associated metabolites, for which plasma levels were available. Seven of these 10 metabolites showed significant association (*P* < 0.05) with at least one of the three cognitive scores (Supplementary Table 4), including ergothioneine (CRAFTDRE: 0.221, *P* = 4.50 × 10^−3^; NACCMOCA: 0.019, *P* = 2.36 × 10^−2^), 2-naphthol sulfate (CRAFTDRE: −0.060, *P* =< 1.00 × 10^−4^; UDSBENTD: −0.049, *P* = 2.90 × 10^−3^; NACCMOCA: −0.055, *P* = 4.00 × 10^−4^) and uridine (CRAFTDRE: 0.009, *P* ≤ 2.37 × 10^−2^; UDSBENTD: 0.013, *P* = 2.60 × 10^−2^; NACCMOCA: 0.013, *P* = 1.00 × 10^−3^) and the four metabolites with yet unknown chemical structure. Thus, all 14 metabolites from the discovery analysis showed significant association (*P* < 0.05) in at least one of the four tests (general cognition in the RSI-4 cohort; CRAFTDRE, UDSBENTD and NACCMOCA in the AGMP cohort) performed for replication.

**Fig. 2:**
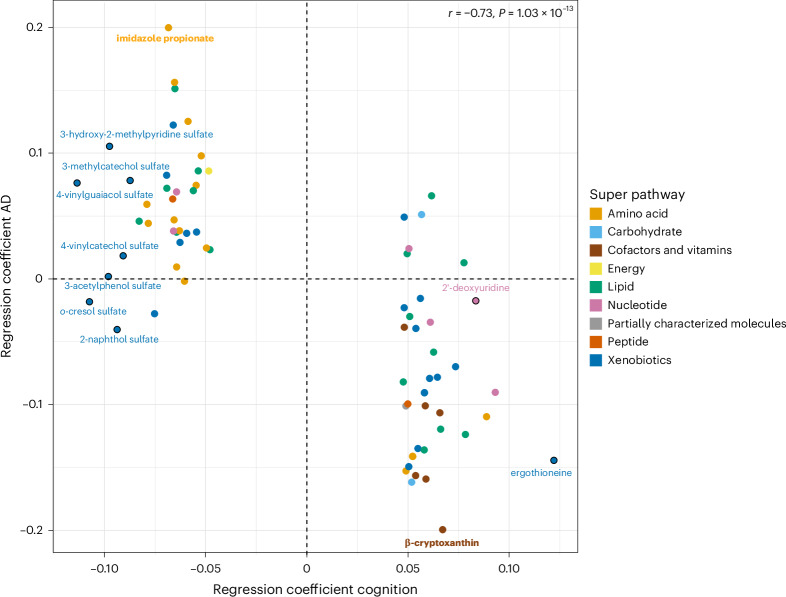
Concordance of metabolite signatures between cognition and incident AD. Scatter plot comparing the regression coefficients of metabolites nominally associated (*P* < 0.05, no multiple testing correction) with general cognition (*x* axis) in the RSIII-2 cohort with their coefficients for incident AD (*y* axis, Cox regression) in the older RSI-4 cohort. Each point represents one metabolite, where colors indicate the classes of metabolites. The nine cognition-associated metabolites that were replicated in RSI-4 are labeled and highlighted through a black border. Additionally, the two cognition-associated metabolites that showed the lowest (β-cryptoxanthin) and highest (imidazole propionate) coefficient in the Cox regression analysis for incident AD are shown in bold. The Pearsonʼs correlation between cognition and AD coefficients is shown (*r* = −0.73, two-sided *P* = 1.03 × 10^−13^). Source data

We additionally compared the regression coefficients of metabolites associated cross-sectionally with general cognition in RSIII-2 (*P* < 0.05) to the Cox regression coefficients observed for the association of these metabolites with incident AD diagnosis during longitudinal follow-up in the independent RSI-4 cohort (*n* = 1,229 (full sample); Table 1) and thereby found a significant correlation between the signals (*r* = −0.73, *P* = 1.03 × 10^−13^) (Fig. 2 and Supplementary Table 4). Among the cognition-related metabolites (*P* < 0.05), β-cryptoxanthin and imidazole propionate showed the largest effects with incident AD diagnosis (β-cryptoxanthin: −0.199, *P* = 3.45 × 10^−4^; imidazole propionate: 0.200, *P* = 3.63 × 10^−4^).

### Sex-stratified analysis suggests association of bile acids with brain health in females

Exploring potential sex differences in metabolite associations, we found suggestive evidence of interaction with sex (*P*_interaction_ < 0.05) for 22 and 61 metabolites, in their association with general cognition and MRI markers, respectively (Supplementary Table 5). We further examined these metabolites in sex-stratified analyses (females, 491; males, 407). Lower blood levels of five metabolites, including taurocholate and glycocholate, were significantly (FDR < 0.05) associated with better general cognition only in female participants, whereas higher levels of one metabolite (N-acetyl-aspartyl-glutamate (NAAG)) were associated with better cognition only in male participants. Analogous analysis for the three MRI phenotypes also revealed more female-specific than male-specific associations (*n* = 21 versus *n* = 6; Supplementary Table 5). Interestingly, among the six female-specific metabolites associated with hippocampal volume, five were conjugated bile acids (glycocholate, taurocholate, glycochenodeoxycholate, taurochenodeoxycholate and taurodeoxycholate) with lower levels being associated with larger hippocampal volume. However, none of these sex-stratified findings replicated in the RSI-4 cohort (Supplementary Table 5).

### Exposome has strongest influences on metabolites associated with brain health

To investigate which unmodifiable and potentially modifiable factors influence the blood levels of the 36 metabolites that associated with general cognition or MRI markers, we estimated how much of each metabolite’s variance is explained by genetic (single-nucleotide polymorphisms (SNPs)), lifestyle (BMI, alcohol consumption, smoking and education), clinical (diabetes and blood pressure), medication (*n* = 31) and microbial (based on 16S rRNA sequencing data) features in the RSIII-2 cohort (Methods and Supplementary Table 6). For genetic factors, we chose a conservative approach by only considering SNPs that showed genome-wide significant associations (*P* = 5.0 × 10^−8^) with metabolite levels in this cohort. For comparison with genetics-based explained variance (EV), SNP-based heritability estimates are provided in Supplementary Table 7.

**Fig. 3:**
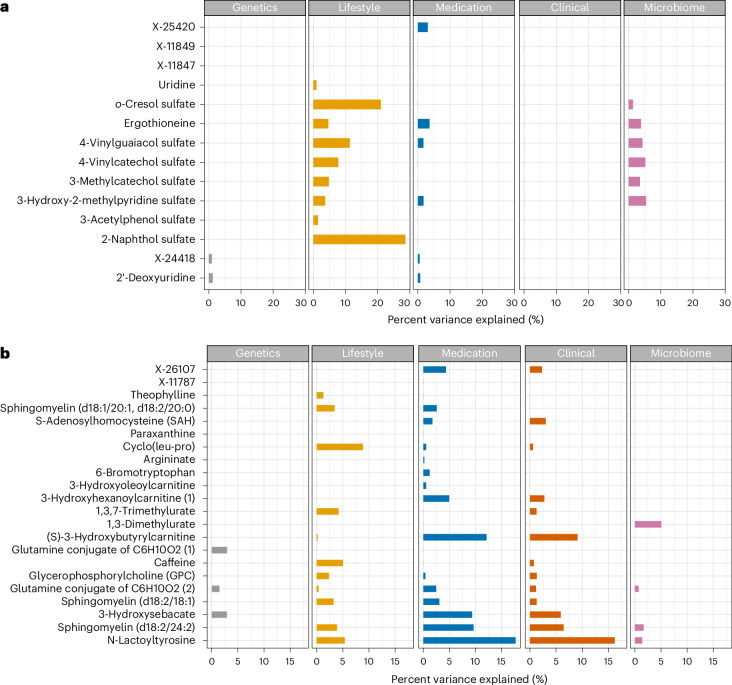
Variance of brain health-associated metabolites explained by genes, gut microbiome and exposome. Percentage of EV of general cognition-associated (**a**) and MRI marker-associated (**b**) metabolites by genetics, gut microbiota, lifestyle, clinical variables and medication use. Note that N-lactoyltyrosine is the updated annotation of a metabolite that was annotated as 1-carboxyethyltyrosine in the original dataset. This correction was provided by Metabolon. Source data

**Extended Data Fig. 3:**
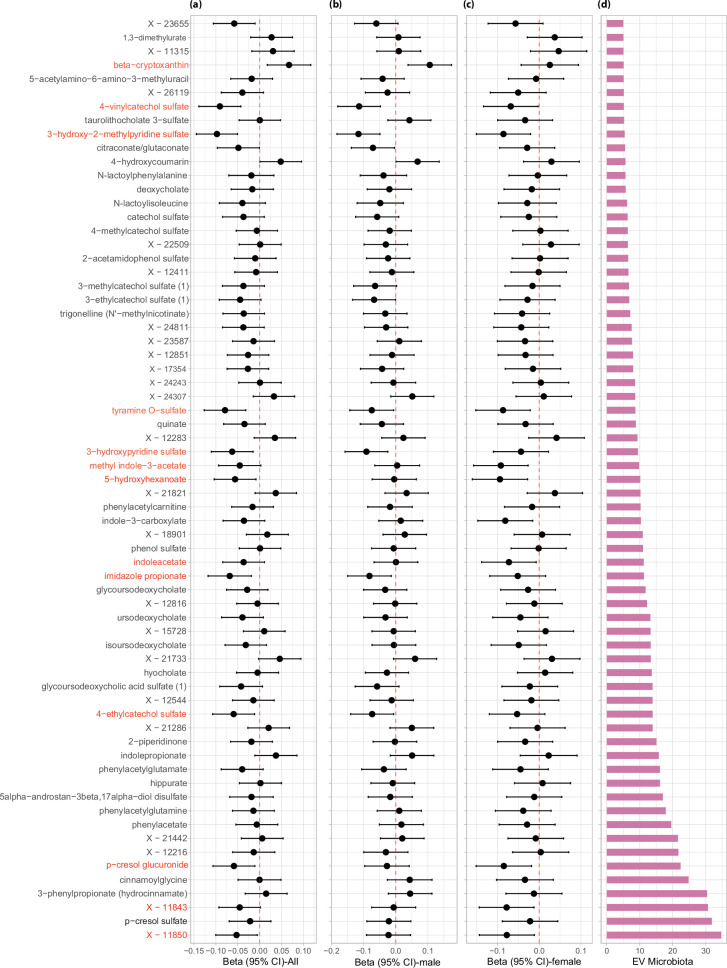
Associations of microbiome-influenced metabolites with general cognition in all, male, and female participants. Forest plot showing the associations of metabolites strongly influenced by the gut microbiome (explained variance (EV) ≥ 5%) with general cognition in all (**a**), male (**b**) and female (**c**) participants of the RSIII-2 cohort. Points represent β estimates from the linear regression models, and horizontal bars represent 95% confidence intervals (CI). Metabolites associated with general cognition at nominal significance (two-sided *P* value < 0.05) are shown in red. (**d**) Bar plot showing the percentage of EV of metabolite levels by gut microbiota for the metabolites displayed in panels **a**–**c**. Source data

Among the metabolites associated with general cognition, lifestyle features explained considerable parts of the variance of nine metabolites, including 2-naphthol sulfate (28.6%), *o*-cresol sulfate (21.0%), 4-vinylguaiacol sulfate (11.3%), 4-vinylcatechol sulfate (7.8%) and ergothioneine (4.7%) (Fig. 3a). Genetics explained 1.2% of the variance of 2′-deoxyuridine and 0.9% of X-24418. Although clinical factors did not account for the variance observed for any of the 14 cognition-associated metabolites, medication did explain part of the variance for six metabolites, with the highest value being observed for ergothioneine (3.6%). Gut microbiota also explained part of the variance for six of the cognition-associated metabolites, including 3-hydroxy-2-methylpyridine sulfate (5.4%), 4-vinylguaiacol sulfate (4.3%) and ergothioneine (3.9%). Interestingly, we observed higher influence of the gut microbiome for the metabolites that showed largest effect sizes in the analysis of incident AD diagnosis in the older RSI-4 cohort. For example, the gut microbiome explained more than 5% of the variance in measured blood levels of various bile acids and the cognition-related metabolites (*P* < 0.05) imidazole propionate (11.3%) and β-cryptoxanthin (5.1%) (Supplementary Table 6 and Extended Data Fig. 3).

For metabolites associated with total brain volume or WML volume, lifestyle (11 metabolites; EV range, 0.20−8.8%), medication use (15 metabolites; EV range, 0.20−17.7%) and clinical factors (13 metabolites; EV range, 0.6−16.2%) were the major factors influencing the metabolites’ variance. Gut microbiota explained part of the variance of four metabolites (0.7−5.1%). Genetic factors explained some of the variance of three metabolites (1.5−2.9%) (Fig. 3b and Supplementary Table 6).

**Fig. 4:**
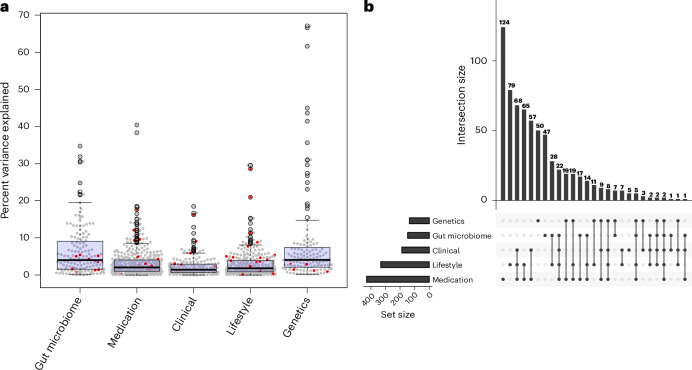
Overall comparison of metabolite variance explained by genes, gut microbiome and exposome. **a**, Box plot showing the percentage of EV of all 991 investigated metabolites by gut microbiome (*n* = 922 participants), medication use (*n* = 1,068 participants), clinical variables (*n* = 1,054 participants), lifestyle factors (*n* = 1,054 participants) and genetics (*n* = 925 participants). Each point represents an individual metabolite out of the 991. Box plots show the median EV (center line), IQR (box; 25th−75th percentiles) and whiskers extending to 1.5 times the IQR; points beyond the whiskers represent outliers. Red dots represent metabolites associated (FDR < 0.05) with general cognition or with MRI markers in our analysis. **b**, UpSet plot showing the overlap of metabolites with EV > 0 by the five tested classes of features (genetics, gut microbiota, lifestyle, clinical variables and medication use). Source data

Next, we compared the influences on the cognition-related and MRI-related metabolites with those observed for all profiled metabolites (Fig. 4 and Supplementary Table 6) and tested whether the associated metabolites were enriched in the set of metabolites with EV > 0 for any factor. Of the 991 tested metabolites, genetics explained some variance (maximal 67.0%) for 130 metabolites. Gut microbiota contributed to the levels of 148 metabolites, explaining up to 34.7% of the variance. Thereby, estimates for the EV of metabolite levels from our study were largely consistent with the ones reported in Bar et al.^25^ and Chen et al.^26^ for genetic and gut microbiome determinants, considering overlapping metabolites (Supplementary Table 8). A detailed comparison of EV results is provided in the Supplementary Text. Lifestyle factors explained up to 29.6% of the variance for 333 metabolites, clinical features explained up to 18.4% for 188 metabolites and medication use explained up to 40.3% for 430 metabolites. Among the five tested feature groups, EV values (EV > 0) of metabolites for clinical features showed a strong positive correlation (FDR < 0.05) with those for medication use (*r* = 0.660) and lifestyle factors (*r* = 0.202) (Supplementary Fig. 3).

For the 36 cognition-related and MRI-related metabolites, significant enrichment (considering the EVs of all 991 studied metabolites) was observed for the influence of lifestyle factors and clinical features, with 20 metabolites being explained to some extent by lifestyle factors (*P* = 6.48 × 10^−3^) and 13 metabolites by clinical features (*P* = 1.48 × 10^−2^). We did not observe significant enrichment for medication use or genetics (medication use: 21 metabolites, *P* = 8.56 × 10^−2^; genetics: five metabolites, *P* = 0.803). With 10 cognition-related or MRI-related metabolites, for which the variance was partially explained by gut microbiota, the enrichment narrowly missed the significance threshold (*P* = 5.15 × 10^−2^).

### Link of brain health-associated metabolites and individual microbial or exposomal features

To further disentangle the determinants of metabolites associated with cognition and MRI phenotypes, we checked their association with lifestyle factors, clinical features, medication use and gut microbiota in univariate regression analyses. The complete results are provided in Supplementary Tables 9–12.

**Extended Data Fig. 4:**
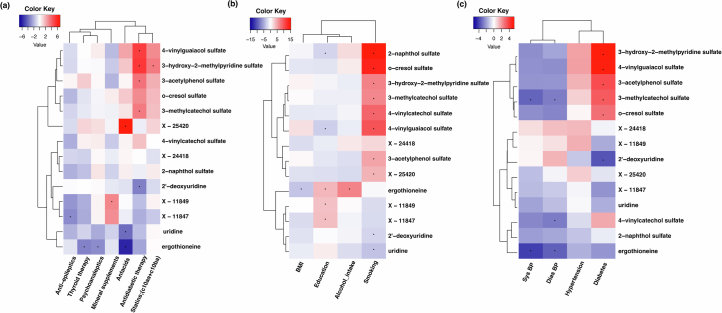
Association of cognition-associated metabolites with medication use, lifestyle, and clinical factors. Heatmap showing the relationship of cognition-associated metabolites with medication use (**a**), lifestyle (**b**), and clinical factors (**c**) in univariate linear regression analysis. Colors represent the direction and magnitude of *z*-value (regression coefficient divided by its standard error), with red indicating positive associations and blue indicating negative associations. Asterisks (*) in the heatmap represents a significant association at FDR < 0.05 based on the association between all tested metabolites with each set of tested features. Source data

Of the 14 cognition-associated metabolites, 13 showed significant association (FDR < 0.05) with different lifestyle factors (Extended Data Fig. 4 and Supplementary Table 9). In particular, smoking was associated with higher levels of the sulfated metabolites (for example, *o*-cresol sulfate and 3-methylcatechol sulfate) and X-25420 and with lower levels of uridine and 2′-deoxyuridine (that is, matching the association pattern of worse cognition). Ergothioneine was associated with all tested factors except smoking; thereby, higher blood levels of ergothioneine, which were linked to better cognition, were associated with higher alcohol intake and higher education and with lower BMI.

Among the tested clinical factors and medications, diabetes and antidiabetic medication were the factors with the highest number of associations (Extended Data Fig. 4 and Supplementary Tables 10 and 11). Higher levels of five of the seven sulfates (including 3-methylcatechol sulfate) and lower levels of 2′-deoxyuridine were significantly associated with diabetes and, apart from *o*-cresol sulfate, also with antidiabetic medication. Interestingly, higher levels of 3-methylcatechol sulfate, which were associated with worse cognition, were associated with lower systolic and diastolic blood pressure. Conversely, higher levels of ergothioneine linked to better cognition were associated with lower blood pressure. Antacids, thyroid therapy and psychoanaleptics were associated with lower blood levels of ergothioneine, with largest effect being observed for antacid use (−0.45, *P* = 4.27 × 10^−9^).

**Extended Data Fig. 5:**
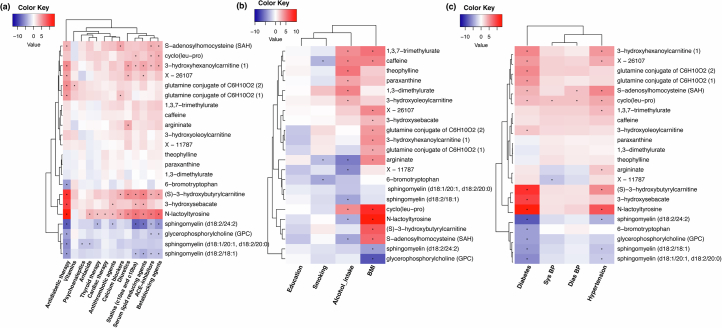
Association of brain imaging marker-associated metabolites with medication use, lifestyle and clinical factors. Heatmap showing the relationship of magnetic resonance imaging (MRI) marker-associated metabolites with medication use (**a**), lifestyle (**b**), and clinical factors (**c**) in univariate linear regression analysis. Colors represent the direction and magnitude of the *z*-value (regression coefficient divided by its standard error), with red indicating positive associations and blue indicating negative associations. Asterisks (*) in the heatmap represents a significant association at FDR < 0.05 based on the association between all tested metabolites with each set of tested features. Note that N-lactoyltyrosine is the updated annotation of a metabolite that was annotated as 1-carboxyethyltyrosine in the original data set. This correction was provided by Metabolon. Source data

Although smoking was the major lifestyle factor associated with metabolites linked to cognition, alcohol intake and BMI showed the highest number of associations observed for the metabolites linked to MRI phenotypes, with 20 of the 22 metabolites being associated with BMI and/or alcohol intake (Extended Data Fig. 5b and Supplementary Table 9). Thereby, higher levels of seven metabolites, of which six are known to be linked to coffee intake and the caffeine pathway, and lower levels of five metabolites (including SAH and N-lactoyltyrosine) were associated with higher alcohol intake. Six metabolites that were associated with alcohol intake were also associated with BMI but three of them in the opposite effect direction (N-lactoyltyrosine, argininate and SAH). Regarding clinical factors and medication, most associations were observed for diabetes, hypertension and antidiabetic medication (Extended Data Fig. 5a,c and Supplementary Tables 10 and 11). The association patterns largely overlapped and resembled the effect directions of worse brain health and higher BMI. Except five metabolites linked to the caffeine pathway, all MRI-associated metabolites were associated with some medication, of which the association of antidiabetic therapy and N-lactoyltyrosine showed the lowest *P* value (adjusted mean difference = 1.54, *P* = 2.84 × 10^−36^).

**Extended Data Fig. 6:**
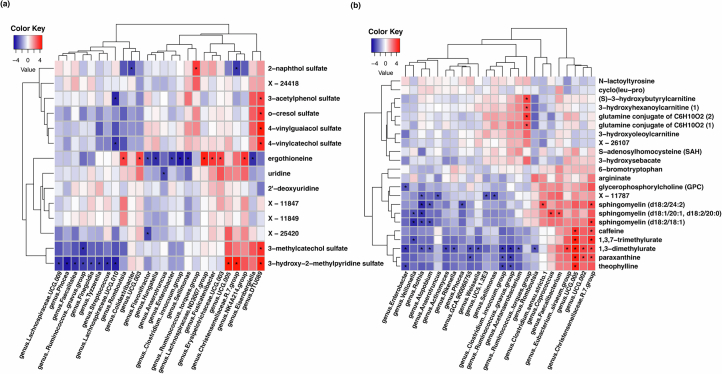
Associations of cognition-associated and brain imaging marker-associated metabolites with gut microbiota. (**a**) Heatmap showing the association between cognition-associated metabolites and gut microbiota at the genus level. (**b**) Heatmap showing the association between magnetic resonance imaging (MRI) marker-associated metabolites and gut microbiota at the genus level. Colors represent the direction and magnitude of the z-value (regression coefficient divided by its standard error), with red indicating positive associations and blue indicating negative associations. Asterisks (*) in the heatmap represents a significant association (FDR < 0.05) based on the association between all tested metabolites with all microbial taxonomic levels. Note that N-lactoyltyrosine is the updated annotation of a metabolite that was annotated as 1-carboxyethyltyrosine in the original data set. This correction was provided by Metabolon. Source data

Of the 36 metabolites associated with general cognition and MRI phenotypes, 22 showed significant association (FDR < 0.05, considering all metabolites (*n* = 991) and microbial features) with specific gut microbiota (Supplementary Table 12 and Extended Data Fig. 6a,b). For example, ergothioneine was associated with 12 microbial genera, with higher levels being associated with a higher abundance of six microbial genera, including *Lachnospiraceae ND3007*, *Fusicatenibacter*, *Romboutsia* and *Erysipelotrichaceae UCG-003*. Higher abundance of genus *DTUO89* was associated with higher levels of six sulfate-containing metabolites, of which three showed lower levels if *Lachnospiracea UCG-010* was more abundant. Among the MRI-associated metabolites, higher levels of (S)-3-hydroxybutyrylcarnitine, 3-hydroxyhexanoylcarnitine and two glutamine conjugates of C6H10O2 were associated with increased abundance of genus *Ruminococcus torques* group. Likewise, higher levels of sphingomyelins were linked to higher abundance of three genera (*Clostridium sensus Stricto 1*, *Coprococcus* and *Faecalibacterium*) and lower abundance of six genera (*Veillonella*, *Rothia*, *Atopobium*, *Phocea*, *Sellimonas* and *UC5-1-2E3*). Levels of the caffeine-related metabolites were associated with 16 microbial genera (Extended Data Fig. 6).

### Ergothioneine mediates the association between antacid medication and cognition

**Fig. 5:**
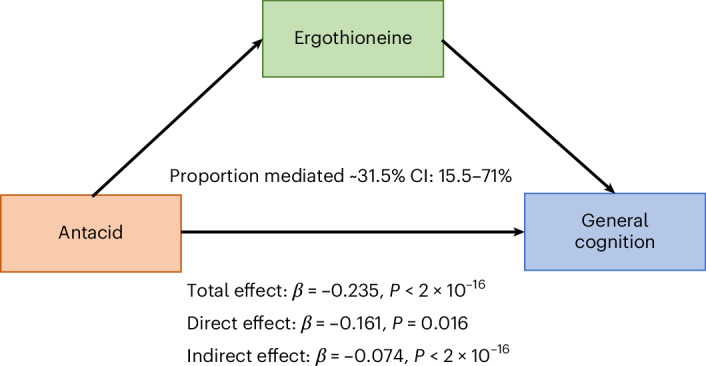
Mediation analysis for the relationship of antacid use, ergothioneine and general cognition. Total, direct and indirect effects were estimated using mediation analysis (R package ‘mediation’) based on linear regression models for the mediator and outcome (*n* = 898). 95% confidence intervals (CIs) were estimated using bootstrap resampling with 500 simulations. Reported (raw) *P* values are two-sided. ~, approximately.

Among our findings, we selected two examples for studying the interplay of brain health, metabolism and its influencing factors in more detail. First, ergothioneine, which robustly associated with general cognition and incident AD, also associated with features of the gut microbiome and the exposome (Supplementary Fig. 4), including a strong association between antacid use and lower blood levels of this metabolite. Also, in the AGMP replication cohort, lower ergothioneine levels were associated (effect size = −0.159, *P* < 0.002) with intake of proton pump inhibitors (PPIs), a common class of antacid medication. Considering recent reports of associations between the use of PPIs and dementia, we investigated the potential mediating role of ergothioneine in the RSIII-2 cohort and found evidence for 31.5% (95% confidence interval: 15.5−71%) of the total (negative) effect of antacid medication on cognition (−0.235, *P* < 2 × 10^−16^) being mediated by ergothioneine (Fig. 5 and Supplementary Table 13).

**Extended Data Fig. 7:**
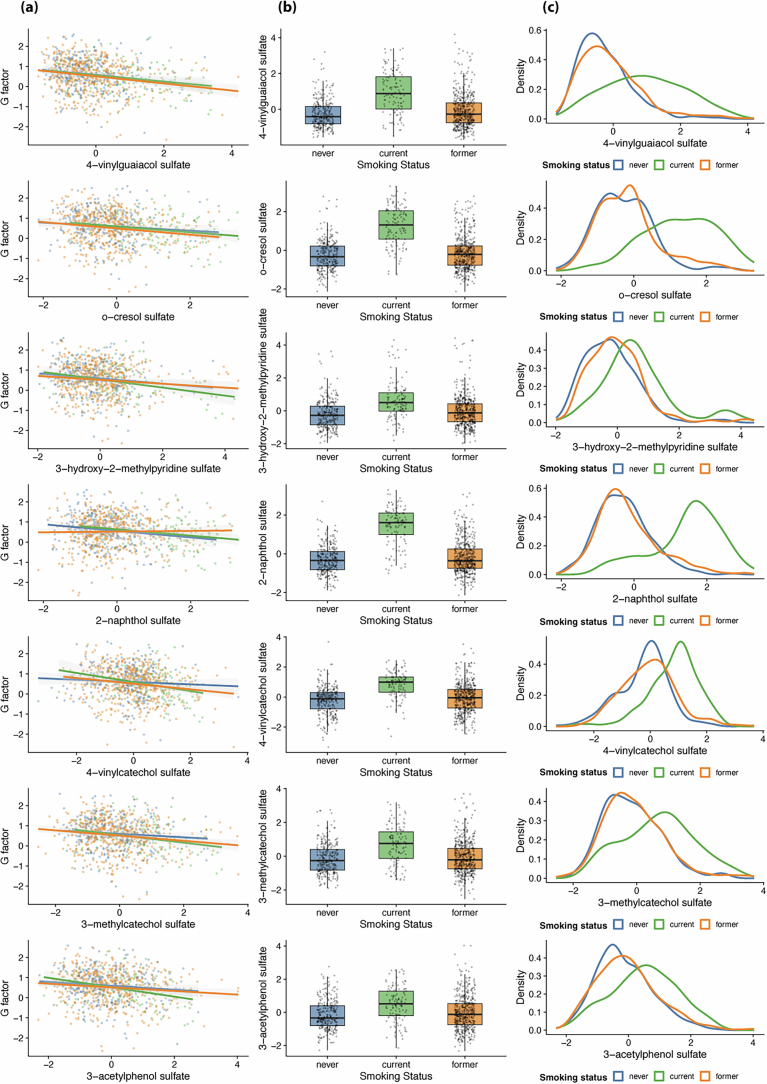
Relationship of sulfated xenobiotic metabolites with general cognition and smoking status. (**a**) Scatter plots showing associations between metabolite levels (*z*-transformed) and general cognition. Each point represents one participant, colored by smoking status (never (blue), current (green), former (orange)). Lines represent fitted linear regression models. (**b**) Box plots showing the distribution of metabolite levels (*z*-transformed) by smoking status. Box plots show the median levels (center line), interquartile range (box; 25th - 75th percentiles), whiskers extending to 1.5 times the interquartile range, and points beyond the whiskers representing outliers. (**c**) Kernel density plots showing the distribution of metabolite levels by smoking status.

As a second example, we further investigated our findings regarding the seven sulfated xenobiotics that associated with general cognition and smoking status. As expected, blood levels of those xenobiotics were consistently higher among current smokers (*n* = 180) compared to never smokers (*n* = 350) and former smokers (*n* = 574) (Extended Data Fig. 7). Stratification on smoking status (Supplementary Table 14) showed that the associations for 3-methylcatechol sulfate (effect size = −0.132, *P* = 4.96 × 10^−2^), 4-vinylcatechol sulfate (−0.140, *P* = 3.66 × 10^−2^), 3-acetylphenol sulfate (0.183, *P* = 5.70 × 10^−3^) and 3-hydroxy-2-methylpyridine sulfate (−0.186, *P* = 5.87 × 10^−3^) remained associated with general cognition (*P* < 0.05) in the group of current smokers. 4-vinylcatechol sulfate (−0.066, *P* = 4.14 × 10^−^^4^), 4-vinylguaiacol sulfate (−0.072, *P* = 3.05 × 10^−2^) and *o*-cresol sulfate (−0.083, *P* = 9.94 × 10^−3^) showed association with cognition in former smokers. In the case of 3-hydroxy-2-methylpyridine sulfate, of which the variance was more explained by gut microbiota (EV 5.4%) than by lifestyle (EV 3.7%), the association with cognition was still significant within the strata of never smokers (−0.118, *P* = 3.42 × 10^−3^).

## Discussion

In this study, we provide an integrated evaluation of how circulating metabolites relate to brain health in midlife and how these metabolites are shaped by modifiable exposures across the exposome. By combining a metabolome-wide analysis of cognition and MRI markers with a systematic assessment of genetic, microbial, lifestyle, clinical and medication determinants of metabolite levels in the population-based Rotterdam Study, we were able to delineate potential metabolic routes through which modifiable factors may influence early brain alterations relevant to AD. Notably, our findings highlight ergothioneine as a metabolite strongly linked to cognition and incident AD risk and reveal that antacid medication may be a relevant determinant of its circulating levels. Although not proving causality, mediation analysis suggests that reduced ergothioneine may partly convey the adverse cognitive association observed with antacids, offering a potentially actionable route through which medication use could affect brain health, if confirmable in longitudinal studies.

Specifically, we observed significant association of 14 metabolites with general cognition and 22 metabolites with MRI markers in middle-aged participants without dementia. For all 14 cognition-associated metabolites, the cross-sectional effects replicated in at least one of two independent cohorts, which included older participants. The effects that we saw for cognition in dementia-free participants were concordant with those for the imaging markers and with the risk estimates for future AD diagnosis, confirming that the metabolite signature derived for cognition in participants prior to any symptoms of dementia is of relevance to understand early metabolic changes in AD etiology. Variance in these metabolites was primarily explained by lifestyle, clinical factors and medication use, with smaller contributions from genetics and the gut microbiome.

To further disentangle the complex relationship of the cognition-associated and MRI marker-associated metabolites with potentially modifiable factors of the exposome or the gut microbiome, we provide a thorough overview of metabolite associations with the single lifestyle, medication, clinical and gut microbial features. Integrating these associations, which can inform about metabolite origin, with metabolite associations to AD-relevant endophenotypes may improve understanding of disease etiology and may help prioritize and identify targets for prevention, as demonstrated for selected examples in the following.

Ergothioneine, a naturally occurring, sulfur-containing amino acid, showed the strongest association with cognition, with higher blood levels relating to better cognition. Consistent with previous work linking ergothioneine to neuroprotection^29^ and reduced cognitive decline^30,31^, our findings support a protective role. Although little is known about the transport of ergothioneine from blood to brain, lower levels in brain were linked to worse cognition, cognitive decline and AD diagnosis in recent studies^32,33^. Microbiome, medication and lifestyle factors explained part of the interindividual variation of blood ergothioneine levels in our study. Although not testable here, diet (for example, mushrooms) also contributes substantially^34^, explaining approximately 14% of the variation of ergothioneine^25^. Moreover, additional factors, including age^35^ and genetics^36^, are known to influence ergothioneine levels. In our study, we additionally found significant associations of antacids, psychoanaleptics, thyroid therapy and systolic and diastolic blood pressure with lower levels of ergothioneine; higher alcohol intake and higher education associated with higher levels. When investigating the relationship of antacid intake with ergothioneine levels further, we found evidence for a mediating role of ergothioneine in the association of antacid with worse cognition in our middle-aged cohort. Antacids such as PPIs are commonly prescribed medications to lower acid concentration in the stomach^37^. In our replication cohort, we confirmed the association of lower ergothioneine levels with PPI intake. However, association between PPI intake and any of the three available cognitive scores was not significant in these older participants. For PPIs, various observational studies reported negative associations with phenotypes of neurodegenerative diseases, whereas other studies did not find such evidence or even reported protective effects^38–40^. Factors such as duration of intake or age have been suggested as modulators of the effect, possibly explaining part of the heterogeneity^40^. Our mediation analysis results suggest the reduction of ergothioneine as one possible mechanism explaining harmful effects of this medication on neurodegenerative diseases. However, this finding should be interpreted cautiously as the temporal sequence of events was assumed for mediation analysis but cannot be ascertained in our cross-sectional dataset where exposure (antacid), mediator (ergothioneine) and outcome (cognition) were assessed at the same visit. Also, it remains unclear whether these medications affect the absorption of ergothioneine from diet (as has been suggested for vitamin B12 (ref. ^41^)) or whether they influence ergothioneine levels by other means. In our study, we also observed significant associations of six microbial genera with lower blood levels of ergothioneine (*Clostridium innocuum* group, *Sellimonas*, *Hungatella*, *Eisenbergiella*, *Enterobacter* and *Flavonifractor*) and six with higher levels. Among the genera linked to lower ergothioneine levels, five belong to the phylum Firmicutes, whose increased abundance has been reported in mild cognitive impairment^42^. *C. innocuum* is a pathogenic bacterium associated with Western high-fat and high-sugar diet^43^. Higher abundance of *Sellimonas* and *Hungatella* have been linked to depression^44^ and *Sellimonas* to atherosclerotic cardiovascular disease^45^; both diseases are risk factors for AD and cognitive decline^46,47^. Infection with *Enterobacter* may also contribute to progression of neurodegeneration^48,49^. Moreover, *Enterobacter* was associated with lower glycerophosphorylcholine, of which higher levels were linked to higher brain volume in our study. In summary, these pieces of evidence point toward a possible role of ergothioneine for maintaining cognitive function and underscore the value of further research into intervention and prevention schemes targeting the management of ergothioneine. Future studies are needed to confirm ergothioneine as a potential mediator of the still-controversial relationship between PPIs and dementia risk.

As a second example, lower levels of seven sulfated xenobiotics, including *o*-cresol sulfate, were associated with better general cognition in our study. These metabolites have been linked to environmental exposures such as smoking^50,51^, and smoking also was the main driver of their variance (except 3-acetylphenol sulfate and 3-hydroxy-2-methylpyridine sulfate) and their association with cognition in our study. However, interestingly, the two sulfated, smoking-associated metabolites, 4-vinylguaiacol sulfate and 4-vinylcatechol sulfate, showed association with cognition in both former and current smokers. Also, the association of 3-hydroxy-2-methylpyridine sulfate with cognition remained significant in never smokers. These findings suggest relevance of those metabolites for AD pathogenesis beyond reflecting smoking behavior, which has been recognized as an important modifiable risk factor of AD previously^52,53^.

Finally, N-lactoyltyrosine, which belongs to a class of pseudopeptides formed by lactic acid and an amino acid^54^, was associated with brain health in our study, with higher levels being linked to lower brain volume. N-lactoyl amino acids received attention in diabetes research recently^55,56^; whereas higher levels, including those of N-lactoylphenylalanine, associate with decreasing glycemic control and incident diabetes, N-lactoylphenylalanine has been shown to reduce appetite in mice^55^ and to mediate the effect of metformin on appetite and weight reduction in humans^57,58^. In our study, N-lactoyl amino acids strongly associated with antidiabetic therapy, confirming these previous findings. Also, glycerophosphorylcholine and hydroxylated acylcarnitines associated with MRI markers resembled the association patterns observed for diabetes and BMI despite adjustment for BMI, emphasizing the shared risk profiles between diabetes and AD. Interestingly, the typical markers of glycemic control, such as glucose or 1,5-anhydroglucitol, were not found associated with MRI markers in our study.

Although the gut microbiome was not prominent in explaining the variance of the cognition-associated and MRI marker-associated metabolites, some microbiome-derived metabolites were linked to cognition and AD risk previously^59–65^, including vitamin A metabolites (for example, β-cryptoxanthin), bile acids (for example, deoxycholate) and indole-derived metabolites (for example, indoleacetate). Interestingly, imidazole propionate and β-cryptoxanthin were the metabolites most strongly associated with the risk of incident AD diagnosis in the older participants of the replication cohort, whereas they showed only nominal significance with general cognition in the younger discovery cohort, suggesting that gut microbiome-related metabolites might be involved later in the pathogenic process. Nonetheless, the generally good concordance of their association effects with cognition in the younger cohort and incident AD in the older cohort indicates a role of the gut microbiome also in asymptomatic phases of AD development, highlighting microbiome-related metabolites and their related microbiota as promising targets for modification through interventions.

One of the main strengths of our study is its dual approach, integrating the identification (and replication) of metabolites linked to midlife brain health with a systematic exploration of the extent to which metabolites are influenced by genetic and potentially modifiable exposomal factors. Beyond the specific incorporation of EV analysis in the study of cognition-related metabolite signatures, our study extends previous phenotype-independent work on EV in metabolomics^25–28^ by adding variance explained by medication use for 991 metabolites, using intake data on 31 common medications in our middle-aged cohort. Notably, in Bar et al.^25^, the limited drug intake by the relatively young and healthy study participants used for EV estimation, resulting in probably underestimated medication-related EVs, was explicitly discussed by the authors as a limitation. However, the EV analysis in our study also comes with various limitations. First, dietary information was not available for the time of blood collection. As diet is known to be an important lifestyle factor defining the levels of many metabolites, this lack of data limits the insights about the impact of the exposome. Also, the impact of physical activity could not be investigated. Second, the estimation of EV strongly depends on the features used as input. For example, applying a less-strict threshold for the inclusion of genetic variants will result in overall higher estimates for the genetic contribution for all metabolites. With the chosen approach, the EVs for genetics were similar to those previously reported by Bar et al.^25^. Also, metabolites driven by gut microbiota showed similar EV percentages when compared to two previous studies^25,26^, underscoring the usability of these EV estimates across studies. Differences in the EV of metabolites by lifestyle and clinical variables with these earlier studies may be attributable to the difference in the inclusion of various features in these types of predictors. Third, clinical features and medication use are inherently connected. As a consequence, we cannot differentiate between the effects of the two factors on metabolite levels within the setting of a population-based cohort.

Although building upon rich data from the Rotterdam Study, our study has further limitations related to data availability. First, MRI measurements were not available exactly at the time of blood and feces collection. We addressed this issue by adjusting for the time difference in our analyses. Moreover, we performed a sensitivity analysis excluding cases for which the time difference exceeded 1 year. Second, our study did not have sufficient power to reliably identify sex-specific metabolite association when considering multiple testing correction. A further limitation is the so-far limited follow-up of the participants of our study with longitudinal cognitive assessments and metabolomics data, which may provide additional insights into the role of metabolites in cognitive decline over time. In the available data on general cognition from 510 RSIII-2 participants, the metabolites cross-sectionally associated with cognition did not show significant association with the change in cognition over time. However, in an earlier cohort of older participants with longer follow-up (RSI-4), effect estimates for cognition−metabolite associations in the cross-sectional analysis significantly correlated with those derived from their Cox proportional hazard ratios for incident AD diagnosis, confirming the relevance of the identified metabolites for future brain health. Also, especially for ergothioneine and uridine, evidence for their relevance in predicting longitudinal cognitive function and AD progression is continuously growing in literature^30,31,66^. Finally, in mediation analyses, we assumed a temporal sequence of exposure, mediator and outcome. However, due to the cross-sectional nature of our analysis, our mediation findings should not be interpreted as evidence of causation. In general, we cannot infer causality through any of the analyses performed in this study. Consequently, the mediating role of ergothioneine warrants confirmation in longitudinal cohorts, where the temporal order of antacid (for example, PPI) intake and the decline of ergothioneine and cognitive performance can be clearly delineated. Also, further mechanistic work is needed before any conclusions on potential clinical implications, such as reconsideration of the recommendations for the prescription of PPIs, can be drawn.

In conclusion, our study provides compelling evidence that lifestyle factors play a major role in shaping blood metabolites associated with general cognition and MRI markers in dementia-free middle-aged participants. The overall concordance of the metabolite signatures identified for cognition and incident AD in independent samples pinpoints the relevance of the underlying metabolites for the disease. Among the potential determinants of these metabolites, smoking emerged as an important lifestyle factor affecting plasma levels of metabolites associated with cognition, and alcohol intake, BMI, diabetes and diabetes medication were linked to MRI marker-associated metabolites. With the example of antacid medication, for which we observed a negative effect on the blood levels of the presumably neuroprotective metabolite ergothioneine, we illustrate the potential of our approach and the here-derived association catalogs for studying the complex interplay among brain health, metabolism and its influences through genetics, the gut microbiome and exposures (lifestyle and medication), potentially informing future work on disease prevention and early interventions.

## Methods

### Study population

#### Rotterdam Study

The Rotterdam Study is a prospective population-based study from the Ommoord district of Rotterdam, The Netherlands. In 1990, the study was initiated with the inclusion of 7,983 partcipants aged 55 years or older (RSI). It was expanded with the addition of a new cohort of 3,011 participants ≥55 years of age (RSII) from 2000 to 2001 and a further cohort of 3,932 participants aged 45 years or older recruited during 2006−2008 (RSIII). All study participants were extensively interviewed and physically examined at their baseline visits and after every 3−6 years. The study has been approved by the Medical Ethical Committee of Erasmus Medical Center and by the Ministry of Health, Welfare and Sport of The Netherlands. Written informed consents were obtained from each study participant to participate and to collect information from their treating physicians^67^. In the present work, we included data from participants of the RSIII cohort collected at the second follow-up (RSIII-2) for which gut microbiota, metabolomics and genetic data were available. We replicated our findings on general cognition in an independent sample from the fourth follow-up of the RSI cohort (RSI-4), which is unrelated to the discovery RSIII cohort (that is, independent recruitment without overlaps in participants). In RSI-4, metabolomics data were available for 874 participants without dementia or stroke diagnosis during follow-up (9.16 ± 3.33 years) and for 355 participants with incident AD. Note that this sample was enriched for incident AD cases and dementia/stroke-free controls. The mean follow-up time between blood collection and onset of AD symptoms was 5.14 years (s.d. = 4.05 years).

#### Assessment of lifestyle, clinical factors and medication intake

In the Rotterdam Study cohorts, information about lifestyle, clinical factors and medication intake was collected using structural interviews, medical records and pharmacy data during multiple visits. Information about lifestyle factors such as smoking, alcohol consumption and educational attainment was collected based on structured home interviews. Smoking data were classified as never, former or current smokers. Educational attainment was assessed at the baseline visit of the Rotterdam Study cohort and categorized into four groups based on the United Nations Educational, Scientific and Cultural Organization (UNESCO) classification: (1) primary education, (2) lower/intermediate general education or lower vocational education, (3) intermediate vocational education or higher education and (4) higher vocational education or university level^68^. In our study, we combined the education categories (1) and (2) into primary education. Alcohol consumption was assessed as part of dietary interviews, and alcohol intake in grams per day was calculated based on the number of drinks multiplied by the average amount of ethanol in one drink of the alcoholic beverage^69^. BMI was calculated based on height and weight (kg m^−^^2^), which were assessed in participants in standing positions without shoes and heavy outer garments. Medical history (clinical factors) and medication intake were compiled based on various sources, including general practitioner records, pharmacy prescription records or a physical examination at the study center. Blood pressure was recorded at the time of the patients’ visit to the study center at the right upper arm in a seated position; the mean of two measurements was recorded. Glucose levels were measured after overnight fasting (8–14 hours); diabetes was defined as fasting serum glucose levels ≥7.0 mmol l^−1^, non-fasting serum glucose levels ≥11.1 mmol l^−1^ and/or the use of antidiabetic medication (Anatomical Therapeutic Chemical (ATC) code A10)^70^.

#### Genotyping and imputations

Blood from the Rotterdam Study participants was collected during the baseline visit of RSIII. DNA was extracted from blood, and genotyping was performed using the 550K, 550K duo or 610K Illumina arrays. During the genotyping quality control for genetic variants, we applied exclusion criteria, including call rate <95%, Hardy−Weinberg equilibrium *P* < 1.0 × 10^−6^ and minor allele frequency (MAF) < 1%. Sample exclusion criteria included excess autosomal heterozygosity, call rate <97.5%, ethnic outliers and duplicates or family relationships. Genotypes were imputed using the Markov Chain Haplotyping (MACH) package and minimac software^71^ to the 1000 Genomes phase 1 version 3 reference panel^72^. Among the 1,068 participants with metabolomics data (after preprocessing), genotyping information was available for 925 participants.

#### Metabolomics profiling

We profiled blood plasma samples of 1,082 participants of the RSIII-2 cohort using the untargeted Metabolon HD4 platform. The resulting dataset includes 1,387 metabolites of different classes (lipids, amino acids, xenobiotics, nucleotides, cofactors and vitamins, peptides, carbohydrates, energy-related metabolites and uncharacterized metabolites). The details of the Metabolon HD4 analytical methods and data extraction procedure were described elsewhere and are briefly summarized in the Supplementary Text. Based on the batch-normalized data as provided by Metabolon, additional preprocessing steps were performed. First, 14 participants for whom the proportion of missing values across metabolites was greater than 5 × s.d. of the mean missingness in all participants were excluded. Then, metabolites with missingness greater than 70% were excluded. For the remaining metabolites, the coefficient of variance of the 64 aliquots of the NIST Standard Reference Material (SRM) 1950 sample, which were measured throughout the experiment, was determined, and metabolites with coefficient of variance greater than 30% were excluded, leaving 1,111 metabolites after the quality control steps. For the present work, we used data on only the 991 frequent metabolites (missingness less than or equal to 30%). After log_2_ transformation, we imputed the missing values applying a *k*-nearest neighbor approach, which has been shown to provide robust imputation for metabolomics data^73^. *z*-transformation (*µ* = 0, s.d. = 1) was applied for each metabolite before the association analysis. A detailed flowchart of quality control and preprocessing steps is provided in Supplementary Fig. 1.

#### Gut microbiome profiling

Detailed information regarding the collection of fecal samples in the RSIII cohort and the subsequent sequencing procedures were described previously^74^. These sequence data were subjected to a specific 16S rRNA profiling pipeline. In short, raw reads were demultiplexed using a custom script to separate sample FASTQ files based on the dual index. Primers, barcodes and heterogeneity spacers were trimmed off using TagCleaner version 0.16 (ref. ^75^). Trimmed FASTQ files were loaded into R (version 4.0.0) with the DADA2 (ref. ^76^) package version 1.18.0. Quality filtering was performed in DADA2 using the following criteria: trim = 0, maxEE = c(2,2), truncQ = 2 and rm.phix = TRUE. Filtered reads were run through the DADA2 amplicon sequence variant (ASV) assignment tool to denoise, cluster and merge the reads. ASVs were assigned a taxonomy from the SILVA version 138.1 rRNA database^77^ using the Ribosomal Database Project naive Bayesian classifier^78^. The resulting data tables were combined into a phyloseq object using phyloseq^79^.

To remove spurious and likely false-positive ASVs, both an abundance and a prevalence filter were applied to the data. ASVs had to contain at least 0.005% of the total reads to remain in the dataset as well as to be present in at least 1% of the samples and were otherwise removed. Samples were also removed based on several other criteria such as being a possible sample swap, ≥8 days in the mail, known duplicates or poor quality control statistics. For this step, samples with fewer than 4,500 reads or those that lost more than 50% of reads in the last steps of the DADA2 quality control (that is, samples with many reads but for which reads were distributed mainly at rare ASVs) were removed from the data. Also, samples with 4,500−6,000 reads that lost more than 20% of reads in the last steps of the DADA2 quality control were excluded. Alpha diversities were calculated based on this filtered phyloseq object. Additionally, a phylogenetic tree was constructed based on the center sequences of each ASV using the phangorn package, and the result was added to the phyloseq object^80^. Finally, ASV IDs were recoded to numerical IDs, ordered on ASV abundance within the population.

#### Assessment of general cognition

A neuropsychological assessment battery was introduced in the Rotterdam Study between 2002 and 2005 for evaluating cognitive function. This battery of tests included the Stroop test (reading, color naming and interference tasks), a letter-digit substitution task (LDST), a categorical Word Fluency Test (WFT), Purdue Pegboard (PPB) tests for both hands individually and combined and a 15-word verbal learning test based on Rey’s recall of words (15-WLT). A composite measure of overall cognitive function known as ‘*g*factor’ was calculated using principal component analysis, as detailed previously^81^. This *g*factor consists of scores from the Stroop interference test, LDST, verbal fluency task, PPB test and 15-WLT delayed recall score.

#### MRI features

MRI scanning has been performed within the Rotterdam Study using a 1.5-Tesla MRI unit equipped with a dedicated eight-channel head coil (Signa HD platform; GE Healthcare). Brain volumetric measurements, including brain volume, WML volume and intracranial volume, were estimated through automated segmentation^82,83^. Left and right hippocampal volumes were obtained using FreeSurfer (version 5.1) and averaged to determine total hippocampal volume. Participants with severe strokes that could potentially affect segmentation were excluded from the MRI marker analysis. Further details regarding MRI scanning and preprocessing can be found elsewhere^84^. In our MRI sample (*n* = 925), the mean interval between blood collection and MRI was 0.86 years (s.d. = 1.54), with a median of 0.18 years (interquartile range (IQR) = 0.97) and a range of 0−7.97 years.

### Statistics and reproducibility

No statistical method was used to predetermine the study sample size; however, the sample size was similar to previous large-scale metabolomics investigations in population-based settings^4–15^. For metabolite profiling, samples were blinded to the metabolomics service provider (Metabolon) and were randomized across analytical plates to minimize batch effects. During metabolomics data preprocessing, samples with a high proportion of missing metabolite measurements were excluded, as detailed in the metabolomics preprocessing workflow (Supplementary Fig. 1). Associations between metabolites and study outcomes were evaluated primarily using linear regression models or Cox proportional hazards models. Gradient boosting decision tree (GBDT) models were used to evaluate the proportion of variance in blood levels of metabolites explained by different classes of features. Detailed descriptions of the statistical analyses, including specific sample sizes, data transformations and coding of variables, type of statistical model and model parameters, covariates, software packages and R libraries, are provided in the corresponding subsections below. Where applicable, multiple testing was controlled using the Benjamini−Hochberg FDR procedure^85^. All analyses were performed using R (versions R 4.1 and 4.5.1) and Python (version 3.8.5).

#### Association of metabolites with general cognition and MRI markers

To evaluate the association of metabolites with general cognition and MRI markers, we performed linear regression analysis. In the association analyses between general cognition and metabolites, we adjusted the models for age, sex, BMI and lipid-lowering medication (model 1) and additionally for education (model 2). In sensitivity analyses, we further adjusted for smoking, hypertension and diabetes (model 3). Among the MRI markers, we selected total brain volume, hippocampal volume and WML volume as brain markers of neurodegeneration and vascular health. Natural log transformation and *z*-transformation (*µ* = 0, s.d. = 1) were applied before the linear regression analysis. Distributions of log-transformed phenotypes and metabolite levels were visually inspected to confirm that distributions were close to a normal distribution, but this was not formally tested. In the linear models, we adjusted for age at blood collection, the time difference between blood collection and MRI scan, sex, BMI, lipid-lowering medications use and intracranial volume (model 1). In model 2, we additionally adjusted for smoking, hypertension and diabetes. In sensitivity analyses of MRI associations, we performed the regression analysis using model 1 after excluding participants with more than 1 year between blood collection and MRI (*n* = 688). Associations were considered statistically significant at FDR < 0.05.

We also performed a longitudinal analysis of the association between baseline metabolite levels and changes in cognition in the discovery (RSIII-2) cohort. Follow-up cognitive assessment was available for 510 participants at the third visit (RSIII-3), with mean follow-up duration of 9.88 years (s.d. = 1.03; range, 7.18–11.77 years). The mean age at baseline for these participants was 61.27 years (s.d. = 4.85). We used linear mixed-effects models with the nlme R package^86^, including participant ID as random intercepts to account for interindividual variation. Follow-up time in years was calculated from the baseline metabolomics visit. Fixed effects included baseline age, sex, BMI, lipid-lowering medication use, follow-up time, metabolite level and their interaction (follow-up time × metabolite). The interaction coefficients estimate the difference in annual cognitive change per 1 s.d. increase in metabolite level.

In addition to the univariate association analyses, we applied elastic net regularization to model general cognition and MRI phenotypes in the RSIII-2 cohort based on all metabolites, using the glmnet package in R^87^. The dataset was randomly divided into 80% for training and 20% for testing. Hyperparameters were optimized through 10-fold cross-validation, using the root mean squared error (RMSE) as the performance criterion. Model performance was reported as the proportion of variance explained for each phenotype. In addition, we provide the sets of metabolites with non-zero *β* coefficients from the elastic net models as multivariate metabolic signatures for the respective traits.

#### Sex-stratified association analysis

To evaluate the sex-specific association of metabolites with general cognition and MRI markers in RSIII-2, we introduced an interaction term (sex × metabolite) in model 1. Metabolites showing evidence of interaction (*P* < 0.05) were further examined in sex-stratified analyses, adjusting for age, BMI and lipid-lowering medication use. The findings of the sex-stratified association of metabolites with general cognition were further replicated in RSI-4. We performed a power calculation based on post hoc interaction analysis in cognition using non-central *t*-distribution and the interaction term estimate and standard error of our top metabolite, NAAG. Although the power to detect a sex interaction effect of this size was approximately 80% at a nominal *α* = 0.05, the estimated power dropped to approximately 10% when applying Bonferroni correction for testing 991 metabolites.

#### Replication of association results in the RSI-4 cohort

We replicated our association results of metabolites with general cognition in a dementia-free and stroke-free sample (*n* = 874) of RSI-4. We used linear regression adjusting for age, sex, BMI and lipid-lowering medication (model 1), with additional adjustment for educational attainment (model 2) and further adjustment for smoking, hypertension and diabetes in model 3. The MRI measurements were not available for this sample.

#### Replication of association results in the AGMP study

For further replication of the cognition-associated blood metabolites, we assessed their association with three cognitive scores in individuals who were recruited into the AGMP through participating ADRCs across the United States. The AGMP ADRC study is a multi-institution collaborative research initiative to define how interconnected factors, such as the exposome, diet, lifestyle, gut microbiome and AD genotypes, influence the metabolome (https://alzheimergut.org/). Written consent for study participation was obtained by each ADRC under institutional review board review and approval. All study procedures were in accordance with the Declaration of Helsinki and allowed deidentified data to be shared among preapproved researchers. Plasma levels of the tested metabolites were available for 512 participants (mean age 72.2 ± 7.81 years; mean BMI 27.3 ± 5.38 kg m^−^^2^; 61% females; 73% normal cognition, 9% dementia; 79% White, 19% African American, 2% Asian) from seven ADRCs. The levels were determined using the same metabolomics approach as applied in the Rotterdam Study. We tested the metabolites’ associations with the CRAFTDRE (mean = 15.6, s.d. = 4.68), UDSBENTD (mean = 10.5, s.d. = 3.86) and NACCMOCA (mean = 25.2, s.d. = 4.17).

Linear regression models were used to assess the association of metabolites with the three cognitive outcomes and the association of ergothioneine with PPI intake while adjusting for age, sex, BMI, *APOE* genotype and intake of lipid-lowering medication. Missing BMI data (approximately 10%) were imputed based on data from lipidomics and Nightingale Health platforms.

#### Association of metabolites with incidence of AD

To evaluate the relationship of blood metabolites with incidence of AD prospectively, we performed Cox proportional hazard analysis adjusted for age, sex, BMI and lipid-lowering medication use in RSI-4 participants, where metabolomics data for 355 participants with incident AD and 874 controls without dementia during follow-up were available. Mean follow-up time of participants with incident AD was 5.14 years (s.d. = 4.05 years).

#### Association of metabolites with gut microbial and exposomal features

For the association analyses between gut microbiota and circulating metabolites, we performed central log transformation (CLR) on each of the taxonomic levels of the gut microbiome dataset, including phylum, class, order, family, genus and species, using the microbiome package^88^. We performed linear regression analysis to evaluate the association between the plasma levels of metabolites (*z*-transformed) and gut microbial taxa, correcting for effects of age, sex, BMI, medication use (PPIs, metformin, lipid-lowering medication and antibiotics), lifestyle factors (smoking and alcohol intake) and technical covariates such as DNA extraction batch, sequencing batch and time of feces in the mail. We also performed linear regression analysis to evaluate the association of individual features included in medication use (31 medications), lifestyle (BMI, alcohol consumption in grams per day, smoking and education level) and clinical factors (diabetes, hypertension, diastolic blood and diastolic blood pressure), using metabolites as outcome variable. All analyses were adjusted for age at blood collection for metabolomics and sex. We applied the significance threshold of 5% FDR in each set of tested features separately.

#### EV of metabolites

To calculate the EV of 991 circulating metabolites by genetics, gut microbiota, medication use, lifestyle and clinical features, we used the GBDT algorithm from LightGBM (version 2.1.2). We thereby adopted the approach described in Bar et al.^25^. For each group of features, we calculated the EV of each metabolite using five-fold cross validation. The coefficient of determination (*R*^2^) × 100 was interpreted as percentage EV of a metabolite. In the EV calculation for gut microbiota, we used the following parameters: learning_rate = 0.005, feature_fraction = 0.2, min_data_in_leaf = 15, metric = l2, early_stopping_rounds = None, n_estimators = 2000, bagging_fraction = 0.8, bagging_freq = 1. To estimate the EV by the remaining features (genetics, medication use, lifestyle and clinical features), we used the parameters as predetermined in the LightGBM package: learning_rate = 0.01, max_depth = 5, feature_fraction = 0.8, num_leaves = 25, min_data_in_leaf = 15, metric = L2, early_stopping_rounds = None, n_estimators = 200, bagging_fraction = 0.9, bagging_freq = 5.

The genetic, medication, clinical and lifestyle components were defined as follows:

##### Genetics

To calculate percentage EV by genetics, we performed a genome-wide association study (GWAS) for each of the 991 metabolites individually, using HASE software^89^. Only SNPs with imputation quality *R*^2^ > 0.3 and MAF > 0.05 were considered. For SNPs with marginal significance of association with any metabolite (*P* < 5 × 10^−8^), we performed clumping using PLINK 1.9 software^90^ with a *P* value threshold of 5.0 × 10^−8^ and a linkage disequilibrium threshold (*r*^2^) of 0.2 in the 500-kilobase region. In total, 415 independent SNPs reached a significance for 991 metabolites. We extracted their dosage information from the genotype imputed data in the Rotterdam Study participants. In the second step, we used GBDT to calculate the percentage EV of each metabolite by genetic features. For this purpose, we only used genetic variant features associated with that particular metabolite (*P* < 5 × 10^−8^) informed by GWAS summary statistics and clumping. We only considered metabolites explained by genetic features with a coefficient of determination (*R*^2^) greater than zero and FDR < 0.05 for the *P* values of the Spearmanʼs correlation coefficient from the GBDT model. In addition, we calculated heritability estimates (*H*^2^) for all 991 metabolites based on the massively expedited genome-wide heritability analysis (MEGHA) method^91^. Due to the small sample size for heritability calculations, we retained heritability estimates of metabolites greater than zero.

##### Medication use

We defined medication intake features based on yes/no information for 31 general medications for which data were recorded in the RSIII-2 cohort. We included only those medications reported to be used by at least 1% of our participants (*n* = 1,068).

##### Gut microbiota

ASV information of all six taxonomic levels, including phylum (*n* = 10), class (*n* = 17), order (*n* = 38), family (*n* = 62), genus (*n* = 190) and species (*n* = 151), were used in 922 participants.

##### Lifestyle

In the EV calculation for lifestyle factors, we considered BMI, alcohol consumption in grams per day, smoking (current, former and never) and education level (lower, middle and high). Lifestyle information was available for 1,054 participants with metabolomics data available.

##### Clinical factors

Common clinical information, including diabetes, hypertension, systolic and diastolic blood pressure, was used. Full information on clinical parameters was available for 1,054 participants with metabolomics data.

#### Mediation analysis between blood metabolite levels and drug intake

To identify the role of drug-associated metabolites as mediators of drug effects on general cognition, we performed mediation analysis using the ‘mediation’ R package. Specifically, we evaluated the role of ergothioneine as mediator in the association between the use of antacids, psychoanaleptics and thyroid therapy and general cognition in the RSIII-2 cohort.

#### Smoking-stratified association of metabolites with general cognition

To evaluate the role of smoking in the association of seven sulfates with general cognition, we performed a smoking-stratified linear regression analysis of these metabolites with general cognition, adjusting for age, sex, BMI, lipid-lowering medication and educational attainment in current smokers, former smokers and never smokers.

### Reporting summary

Further information on research design is available in the Nature Portfolio Reporting Summary linked to this article.

## Supplementary information

Supplementary Text and Figs. 1−4.

Reporting Summary

Peer Review File

Tables 1–14.

## Source data

Source Data Fig. 1 Statistical Source Data.

Source Data Fig. 2 Statistical Source Data.

Source Data Fig. 3 Statistical Source Data.

Source Data Fig. 4 Statistical Source Data.

Source Data Extended Data Fig. 1 Statistical Source Data.

Source Data Extended Data Fig. 2 Statistical Source Data.

Source Data Extended Data Fig. 3 Statistical Source Data.

Source Data Extended Data Fig. 4 Statistical Source Data.

Source Data Extended Data Fig. 5 Statistical Source Data.

Source Data Extended Data Fig. 6 Statistical Source Data.

## Data Availability

Rotterdam Study data (including RSI and RSIII) can be made available to interested researchers upon request. Requests can be directed to data manager F. J. A. van Rooij (f.vanrooij@erasmusmc.nl). We are unable to place data in a public repository due to legal and ethical restraints. Sharing of individual participant data was not included in the informed consent of the study, and there is potential risk of revealing participants’ identities as it is not possible to completely anonymize the data. This is of particular concern given the sensitive personal nature of much of the data collected as part of the Rotterdam Study. ADRC clinical data are available through the NACC at https://www.naccdata.org/. Access requires a NACC data request using https://www.naccdata.org/data-request-process/. ADRC biochemical data will be shared via the AD Knowledge Portal, https://adknowledgeportal.synapse.org/, and requires Synapse registration to download data.

## Code availability

Code is available via the GitHub repository: https://github.com/omics-x/BloodMetabolomics_Genes_Gut_Exposome.git.
