# Supplementary material for: The blood metabolome of brain health in midlife and influences of genes, microbiome and exposome
Source: Nat Aging. 2026 Jun 24;6(7):1452–67. doi: 10.1038/s43587-026-01149-4 (PMC13375541; doi:10.1038/s43587-026-01149-4)
Supplement: Supplementary file 2 — Reporting Summary [file 43587_2026_1149_MOESM2_ESM.pdf]

nature portfolio

Corresponding author(s): Rima Kaddurah-Daouk & Gabi Kastenmüller

Last updated by author(s): Apr 22, 2026

Reporting Summary

Nature Portfolio wishes to improve the reproducibility of the work that we publish. This form provides structure for consistency and transparency in reporting. For further information on Nature Portfolio policies, see our Editorial Policies and the Editorial Policy Checklist.

Statistics

For all statistical analyses, confirm that the following items are present in the figure legend, table legend, main text, or Methods section.

n/a	Confirmed	
☐	☒	The exact sample size (*n*) for each experimental group/condition, given as a discrete number and unit of measurement
☐	☒	A statement on whether measurements were taken from distinct samples or whether the same sample was measured repeatedly
☐	☒	The statistical test(s) used AND whether they are one- or two-sided<br>*Only common tests should be described solely by name; describe more complex techniques in the Methods section.*
☐	☒	A description of all covariates tested
☐	☒	A description of any assumptions or corrections, such as tests of normality and adjustment for multiple comparisons
☐	☒	A full description of the statistical parameters including central tendency (e.g. means) or other basic estimates (e.g. regression coefficient) AND variation (e.g. standard deviation) or associated estimates of uncertainty (e.g. confidence intervals)
☐	☒	For null hypothesis testing, the test statistic (e.g. *F*, *t*, *r*) with confidence intervals, effect sizes, degrees of freedom and *P* value noted<br>*Give P values as exact values whenever suitable.*
☒	☐	For Bayesian analysis, information on the choice of priors and Markov chain Monte Carlo settings
☒	☐	For hierarchical and complex designs, identification of the appropriate level for tests and full reporting of outcomes
☐	☒	Estimates of effect sizes (e.g. Cohen's *d*, Pearson's *r*), indicating how they were calculated

Our web collection on statistics for biologists contains articles on many of the points above.

Software and code

Policy information about availability of computer code

Data collection	No specific software was used for data collection. Code used for the statistical analysis and generation of the Figures is available via the github repository https://github.com/omics-x/BloodMetabolomics_Genes_Gut_Exposome.git
Data analysis	R 4.1, 4.5.1; Python 3.8.5; Plink 1.9

For manuscripts utilizing custom algorithms or software that are central to the research but not yet described in published literature, software must be made available to editors and reviewers. We strongly encourage code deposition in a community repository (e.g. GitHub). See the Nature Portfolio guidelines for submitting code & software for further information.

Data

Policy information about availability of data

All manuscripts must include a data availability statement. This statement should provide the following information, where applicable:

- Accession codes, unique identifiers, or web links for publicly available datasets
- A description of any restrictions on data availability
- For clinical datasets or third party data, please ensure that the statement adheres to our policy

Rotterdam Study data (including RSI and RSIII) can be made available to interested researchers upon request. Requests can be directed to data manager Frank J.A. van Rooij (f.vanrooij@erasmusmc.nl). We are unable to place data in a public repository due to legal and ethical restraints. Sharing of individual participant data was not included in the informed consent of the study, and there is potential risk of revealing participants' identities as it is not possible to completely anonymize the

data. This is of particular concern given the sensitive personal nature of much of the data collected as part of the Rotterdam Study.
ADRC clinical data is available through NACC at https://www.naccdata.org/. Access requires a NACC data request using https://www.naccdata.org/data-request-process/. ADRC biochemical data will be shared via the AD Knowledge Portal, https://adknowledgeportal.synapse.org/ and requires Synapse registration to download data.

Research involving human participants, their data, or biological material

Policy information about studies with human participants or human data. See also policy information about sex, gender (identity/presentation), and sexual orientation and race, ethnicity and racism.

Reporting on sex and gender

We analyzed data from male and female participants. The sex distributions in the study and replication cohorts are provided in Table 1 (Rotterdam Study) or in the Methods (AGMP ADRC Study). We analyzed sex-interaction in the association between metabolites with general cognition/Magnetic Resonance Imaging phenotypes and performed sex-stratified association analysis for those metabolites that showed evidence of interaction (P<0.05).

Reporting on race, ethnicity, or other socially relevant groupings

For discovery analyses, this study included only participants with European ancestry. The replication cohort (AGMP ADRC Study) included participants with multiple ethnicities (79% White, 19% African American, 2% Asian) but was too small to allow analysis stratified by ethnicity.

Population characteristics

We used data from the Rotterdam Study (RS), a prospective population-based study located in the Ommoord district of Rotterdam, The Netherlands. In 1990, the study was initiated with the inclusion of 7,983 subjects aged 55 years or older (RSI). The study was expanded with the addition of a new cohort of 3,011 participants ≥ 55 years of age (RSII) from 2000 to 2001, and a further cohort of 3,932 participants with age 45 years or older recruited during 2006-2008 (RSIII). All study participants were extensively interviewed and physically examined at their baseline visits and after every 3 to 6 years. The study has been approved by the Medical Ethical Committee of Erasmus Medical Center and by the Ministry of Health, Welfare, and Sport of the Netherlands. Written informed consents were obtained from each study participant to participate and to collect information from their treating physicians. In the current work, we included data from participants of the second follow-up of the RSIII cohort (RSIII-2) for which gut microbiota, metabolomics, and genetic data were available (n=1,068). We replicated our findings on general cognition in an independent sample from the fourth follow-up of the RSI cohort (RSI-4). In RSI-4, metabolomics data was available for 874 participants without dementia or stroke diagnosis during follow-up (9.16 ± 3.33 years) and 355 participants with incident AD. Note that this sample was enriched for incident AD cases and dementia/stroke-free controls. The mean follow-up time between blood collection and onset of Alzheimer's disease symptoms was 5.14 years (SD = 4.05 years).
Additional replication was performed in a cohort collected as part of the Alzheimer's Gut Microbiome Project (AGMP) through participating Alzheimer's Disease Research Centers (ADRCs) across the United States (US) (https://alzheimergut.org/). Plasma levels of the tested metabolites were available for 512 participants (mean age 72.2 ± 7.81 years; mean BMI 27.3 ± 5.38 kg/m2; 61% females; 73% normal cognition, 9 % dementia; 79% White, 19% African American, 2% Asian) from seven ADRCs.

Recruitment

The Rotterdam Study started in 1990. All residents aged 55 and older living in Ommoord, a district of Rotterdam, the Netherlands, were invited. Of 10,215 invited inhabitants, 7,983 (78%) agreed to participate in the baseline examination. In 2000, the cohort was extended to invite all residents who turned 55 or moved into the research area. Of the 4,472 invitees, 3,011 (67%) agreed to participate. Follow-up examinations take place every 3 to 6 years. Participation rates of the Rotterdam Study are generally high compared to reported rates from other population-based cohort studies or biobanks.

Ethics oversight

The Rotterdam Study has medical ethics committee approval per the Population Study Act: Rotterdam Study, executed by the Ministry of Health, Welfare and Sport of the Netherlands. Written informed consent was obtained from all participants. In ADRCs cohorts, written consent for study participation was obtained under Institutional Review Board (IRB) review and approval.

Note that full information on the approval of the study protocol must also be provided in the manuscript.

Field-specific reporting

Please select the one below that is the best fit for your research. If you are not sure, read the appropriate sections before making your selection.

☒ Life sciences ☐ Behavioural & social sciences ☐ Ecological, evolutionary & environmental sciences

For a reference copy of the document with all sections, see nature.com/documents/nr-reporting-summary-flat.pdf

Life sciences study design

All studies must disclose on these points even when the disclosure is negative.

Sample size

The current study is based on 1,068 participants from Rotterdam Study III (RSIII) for whom information on gut-microbiota, genomics, metabolomics and demographic variables including BMI was available for the second follow-up. No statistical method was used to predetermine the study sample size; however, the sample size was comparable to previous large-scale metabolomics investigations using observational data in population-based settings.

Data exclusions

For the current work, samples whose missingness in metabolomics data (Metabolon HD4) was higher than 5x the standard deviation from the mean missingness were excluded (N = 14).

Replication	Replication was performed in a sub-cohort of the Rotterdam Study I (RSI), comprising 874 dementia-free participants and 355 participants with incident Alzheimer's disease, for which general cognition and metabolomics data (Metabolon HD4) were available. Further replication was performed in 512 participants of the AGMP ADRC cohort. All 14 cognition-associated metabolites from the discovery analysis showed significant association (P < 0.05) in at least one of the four tests (general cognition in RSI-4 cohort; CRAFTDRE, UDSBENTD, NACCMOCA in AGMP cohort) performed for replication. In contrast, none of the sex-specific findings from the discovery analysis for which we attempted replication was successfully replicated in the RSI-4 cohort.
Randomization	All the participants for gut-microbiome profiling were randomly selected from the Rotterdam Study III (RSIII) and were further profiled for metabolomics. We aimed to control for confounding by adjusting models for age, sex, BMI, education attainment and technical covariates where applicable in regression models. Samples selected for metabolomic analysis in RSI were enriched for incident AD cases. For metabolite profiling, samples were randomized across analytical plates.
Blinding	Not applicable to this observational study as data was collected prospectively prior to the outcomes occurring. These outcomes were later reviewed and adjudicated by a consensus panel that was blinded to the determinant data, and no treatments or interventions were assigned. For metabolite profiling, samples were blinded to the metabolomics service provider (Metabolon).

Reporting for specific materials, systems and methods

We require information from authors about some types of materials, experimental systems and methods used in many studies. Here, indicate whether each material, system or method listed is relevant to your study. If you are not sure if a list item applies to your research, read the appropriate section before selecting a response.

Materials & experimental systems

n/a	Involved in the study
☒	☐ Antibodies
☒	☐ Eukaryotic cell lines
☒	☐ Palaeontology and archaeology
☒	☐ Animals and other organisms
☒	☐ Clinical data
☒	☐ Dual use research of concern
☒	☐ Plants

Methods

n/a	Involved in the study
☒	☐ ChIP-seq
☒	☐ Flow cytometry
☐	☒ MRI-based neuroimaging

Plants

Seed stocks	N/A
Novel plant genotypes	N/A
Authentication	N/A

Magnetic resonance imaging

Experimental design

Design type	Observational study
Design specifications	N/A
Behavioral performance measures	N/A

Acquisition

Imaging type(s): Magnetic resonance imaging

Field strength: 1.5T

Sequence & imaging parameters: T1-weighted, T2-weighted-Fluid-Attenuated Inversion Recovery

Area of acquisition: Brain

Diffusion MRI: ☐ Used ☒ Not used

Preprocessing

Preprocessing software: Global brain volumes were quantified through an in-house segmentation tool described in Vrooman HA, et al. Multi-spectral brain tissue segmentation using automatically trained k-nearest-neighbor classification. Neuroimage. 2007;37(1):71–81. In brief, k-nearest-neighbor classification was automated by non-rigidly registering MR data with a tissue probability atlas. All segmentations were manually checked and corrected if necessary. Total brain volume was calculated as the sum of gray matter, normal-appearing white matter, and white matter hyperintensities (WMH) volume. White matter was the sum of normal-appearing white matter and WMH volumes. Supratentorial intracranial volume, as a proxy for head size, was calculated by summing CSF volumes to the total brain volume. Hippocampal volume was extracted through Freesurfer 6.0.

Normalization: All scans were transformed to the high-resolution data set (256 × 256 × 128) using tri-linear interpolation. Automated processing tools from the Brain Imaging Center, Montreal Neurological Institute and McGill University (www.bic.mni.mcgill.ca) were used to coregister the MRI data (based on mutual information) and subsequently normalize the intensities for each feature image volume using N3.

Normalization template: MNI-152

Noise and artifact removal: N/A

Volume censoring: N/A

Statistical modeling & inference

Model type and settings: Linear regression

Effect(s) tested: Association of metabolites with MRI variables

Specify type of analysis: ☒ Whole brain ☐ ROI-based ☐ Both

Statistic type for inference (See Eklund et al. 2016): *Specify voxel-wise or cluster-wise and report all relevant parameters for cluster-wise methods.*

Correction: False Discovery Rate

Models & analysis

n/a	Involved in the study
☒	☐ Functional and/or effective connectivity
☒	☐ Graph analysis
☐	☒ Multivariate modeling or predictive analysis

Multivariate modeling and predictive analysis: Linear regression analysis was used to associate the blood levels of metabolites with MRI markers adjusted for age, sex, BMI, lipid-lowering medication use and technical covariates. We also performed elastic net regularization models to predict MRI markers based on metabolites and reported the R2 value for the outcome.
