# Supplementary material for: The blood metabolome of brain health in midlife and influences of genes, microbiome and exposome
Source: Nat Aging. 2026 Jun 24;6(7):1452–67. doi: 10.1038/s43587-026-01149-4 (PMC13375541; doi:10.1038/s43587-026-01149-4)
Supplement: Supplementary file 3 — Peer Review File [file 43587_2026_1149_MOESM3_ESM.pdf]

nature portfolio

Peer Review File

The blood metabolome of brain health in midlife and influences of genes, microbiome, and exposome

Corresponding Author: Dr Gabi Kastenmüller

Version 0:

Reviewer comments:

Reviewer #1

(Remarks to the Author)
The blood metabolome of cognitive function and brain health in middle-aged adults
– influences of genes, gut microbiome, and exposome

In the present study, the authors have examined the relation of blood metabolites with general cognition, MRI in 1082 dementia free participants from the Rotterdam study. In addition, they have evaluated how much genetic variation, lifestyle, comorbidities, medication, and gut microbiota explained variance. The study showed that twenty-one metabolites were associated with total brain volume and total white matter lesions. Fourteen metabolites were associated with cognition and they identified that ergothioneine exhibited the largest effect. The study is very well-written, the objectives are clearly defined, the methods are sound, and the conclusions support the results. The supplementary materials are detailed and informative. I commend the authors on their interesting and comprehensive work. I have a few comments that deserve attention.

1. Abstract: The design of the study could be clearer in the abstract.
2. Introduction: The introduction is well-written with supporting references and clear objectives.
Results:
1. The design of the study is not clear at first when reading the results. It should be made clear to the reader that the first analysis evaluates metabolites with cognition and MRI, at the same time point (cross-sectional) if that is the case. For AD it needs to be clear whether this is a case-control or a prospective design. The follow-up needs to be reported in the abstract.
2. For the first aim of evaluating brain MRI with distinct blood metabolites, I am wondering whether the authors have decided to use a hypothesis driven approach to evaluate each metabolite with the outcomes rather than using a data driven approach or machine learning methods (such as elastic net regressions) to identify metabolite signatures of the outcomes.
3. Have the authors considered for this first analysis using a more complete model including other variables such as smoking, hypertension or diabetes.
4. The authors could clarify whether GWAS was conducted for each metabolite individually or in combination.
Discussion
5. It would be good to highlight better the novelty of this work in the discussion as well as the clinical implications.

Reviewer #2

(Remarks to the Author)
Ahmad et al. examined associations between 991 plasma metabolites, cognition, and MRI-derived neuroimaging markers in two Rotterdam Study cohorts. They quantified variance in metabolites attributable to genetics, gut microbiota, lifestyle, clinical factors, and medications. Fourteen metabolites were linked to cognition, with nine replicating in an older cohort and aligning directionally with incident AD. Ergothioneine, identified as neuroprotective, showed reduced levels with antacid use and partially mediated the antacid-cognition relationship.

Major comments
1. The study lacks a clear hypothesis (e.g., biomarker discovery, mechanistic insight, or clinical prediction). Clarify aims and align results presentation accordingly (e.g., include independent replications, mechanistic experiments, or predictive

performance). The current replication population is from part of the discovery population, and therefore, the validity of replication is somehow undermined.
2. Assess whether cognition-associated metabolites predict longitudinal cognitive decline within the Rotterdam cohort to strengthen biomarker relevance.
3. Please report the distribution of blood collection-to-MRI scan intervals, and test whether the significant metabolite-MRI marker associations persist after excluding individuals with long intervals (e.g., >1 year).
4. Mediation assumes a temporal sequence between the exposure, mediator, and outcome, which the manuscript does not seem to establish. A clear study timeline should be provided to justify the mediation analysis. Furthermore, the mediated association (antacids, ergothioneine, and cognition) is novel and interesting, however, the underlying mechanism is unclear based on the current literature. I would tone down the relevant discussion.
5. Clarify if sex-stratified analyses are exploratory and address statistical power for detecting sex-specific effects.
6. I have a concern regarding the novelty of the explained variation analysis, considering the 2022 Nature Medicine article, “Influence of the microbiome, diet and genetics on inter-individual variation in the human plasma metabolome”, which explored similar aspects of exposome and microbiome interactions with the metabolome. The authors should clearly articulate how their findings advance beyond previous work, contrast their variance estimates with those reported in the Nat Med article, and discuss discrepancies.

Minor comments
1. Page 7: Specify the exact annotation for "glutamine conjugates of $C_6H_{10}O_2$ (2)."
2. Nomenclature: Ensure consistency (e.g., "3-methylcatechol sulfate" vs. "3-methyl-catechol sulfate (2)").
3. Figure 2: Clarify labeling criteria for significant metabolites; resolve discrepancy between labeled (10) and described (9) replicated metabolites.
4. Figure S3: Use bounding boxes/shading to group metabolite clusters in the correlation matrix for clarity.
5. Figure S7: Add the missing column header in the heatmap.

Reviewer #3

(Remarks to the Author)
This work present significant originality and significance a it integrates blood metabolomics (991 metabolites) with neuroimaging and cognition data, which is still relatively novel, especially at this scale and depth. It not only identifies associations between metabolites and brain measures but also assesses the sources of variance in metabolite levels, including:
Genetics, Lifestyle, Comorbidities, Medication and Gut microbiota.

Overall, this is a very well written manuscript, with appropriate use of statistics.

In this study, 991 frequent metabolites in 1,068 participants were characterized through the untargeted commercial platform Metabolon in the Rotterdam study III and associated with cognition and brain imaging (pre-AD diagnosis). For cognition, the findings were replicated in 874 participants of the older RSI-4 cohort and in a later follow up with patients with incident AD (355). This study constitutes a large sample size for this type of analysis with replication which re-enforced the findings.

Minor comment:
Results from the sex stratified analyses should be summarized differently. The first step would be to run models with an interaction term between metabolite and sex and stratify in the case of a p-value under 0.05 for example. Then, the authors should report effect sizes in both males and females and not just p-values of individual models.

Version 1:

Reviewer comments:

Reviewer #1

(Remarks to the Author)
The authors have successfully addressed all of my previous comments, as well as the concerns raised by the other reviewers. They have made substantial improvements to the manuscript, including clearer writing, including discussion of limitation, the addition of new suggested analysis (such as the new model 3 in supplemental table 1 with additional adjustments), additional replication, and clarification of sex-stratified analyses as requested by other reviewers. Overall, the manuscript has been significantly strengthened and I have no further comments.

Reviewer #2

(Remarks to the Author)
The authors have addressed all of my comments very thoroughly in the revised manuscript. The quality of the manuscript has improved substantially, and the revisions have strengthened the clarity and overall presentation. I congratulate the authors on these improvements.

Open Access This Peer Review File is licensed under a Creative Commons Attribution 4.0 International License, which permits use, sharing, adaptation, distribution and reproduction in any medium or format, as long as you give appropriate credit to the original author(s) and the source, provide a link to the Creative Commons license, and indicate if changes were made.
In cases where reviewers are anonymous, credit should be given to 'Anonymous Referee' and the source.
The images or other third party material in this Peer Review File are included in the article's Creative Commons license, unless indicated otherwise in a credit line to the material. If material is not included in the article's Creative Commons license and your intended use is not permitted by statutory regulation or exceeds the permitted use, you will need to obtain permission directly from the copyright holder.
To view a copy of this license, visit https://creativecommons.org/licenses/by/4.0/

Response to reviewers' comments

We thank the three reviewers for their valuable comments and suggestions. We thoroughly went through all the points raised and provide our point-by-point responses below. We believe that our manuscript has greatly improved through this revision process.

Reviewer #1

In the present study, the authors have examined the relation of blood metabolites with general cognition, MRI in 1082 dementia free participants from the Rotterdam study. In addition, they have evaluated how much genetic variation, lifestyle, comorbidities, medication, and gut microbiota explained variance. The study showed that twenty-one metabolites were associated with total brain volume and total white matter lesions. Fourteen metabolites were associated with cognition and they identified that ergothioneine exhibited the largest effect. The study is very well-written, the objectives are clearly defined, the methods are sound, and the conclusions support the results. The supplementary materials are detailed and informative. I commend the authors on their interesting and comprehensive work. I have a few comments that deserve attention.

> **Response:** We very much appreciate this positive assessment of our work and the insightful comments of this reviewer, which helped us to substantially improve the quality and clarity of the manuscript.

Comment 1: Abstract: The design of the study could be clearer in the abstract.

> **Response**: To enhance clarity, we rephrased this part in the abstract and incorporated a more detailed description of the study design (**lines 5–11)**:
>
> *"… We examined the relation of 991 blood metabolites with general cognition and magnetic resonance imaging (MRI) cross-sectionally in 1,082 dementia-free middle-aged participants of the population-based Rotterdam Study, and assessed how much genetic variation, lifestyle, comorbidities, medication, and gut microbiota explained metabolite variance. Cognition-associated metabolites were replicated in two independent cohorts of older participants cross-sectionally and were additionally tested for their association with incident AD longitudinally in one of the cohorts. …"*

Comment 2: Introduction: The introduction is well-written with supporting references and clear objectives.

> **Response:** Thank you.

Comment 3: Results: The design of the study is not clear at first when reading the results. It should be made clear to the reader that the first analysis evaluates metabolites with cognition and MRI, at the same time point (cross-sectional) if that is the case. For AD it needs to be clear

whether this is a case-control or a prospective design. The follow-up needs to be reported in the abstract.

Response: Indeed, the associations of the metabolites with cognition and MRI markers were evaluated cross-sectionally, and the associations of metabolites with AD were evaluated longitudinally (using Cox regression) in a prospective design. Thank you for pointing out that this was not fully clear from our description in the original manuscript. To enhance clarity, we have included more detailed information about the study design in the Introduction and the Results section of the revised manuscript. These parts now read:

Introduction, lines 60–66:

"… Specifically, we first investigated the cross-sectional association of circulating metabolites with general cognition and MRI markers in midlife within a dementia-free sample of the population-based Rotterdam Study (RS) to identify potential biomarkers of brain health. The resulting cognition-associated metabolites were replicated cross-sectionally in two independent cohorts of older individuals. To ensure the relevance of these metabolites for AD, they were further tested for their relationship with incident AD diagnosis during follow-up in one of the replication cohorts in a prospective design. In a second step, …"

Results, lines 73–94:

"For this study, the levels of 1,387 metabolites were determined in plasma samples from 1,082 participants of the RSIII-2 cohort (i.e., the third cohort at second follow-up) of the Rotterdam Study using a non-targeted metabolomics approach (Metabolon HD4). To elucidate the connection of the peripheral metabolome with brain health and function, we first investigated the cross-sectional association of general cognition and MRI markers with the levels of 991 frequent metabolites in the 1,068 RSIII-2 participants (after data preprocessing) (see Methods; Supplementary Figure 1). For cognition, we replicated our findings in 874 participants of the older RSI-4 cohort, an independent sample (i.e., separate survey, no overlap with discovery cohort) of the Rotterdam Study, for which general cognition (G-factor) and metabolomics data from the same platform were available at the same time point. The 874 RSI-4 participants used for the replication analysis did not have any diagnosis of dementia (or stroke) at this time or during later clinical follow-ups. As a second replication analysis, we tested the association of the cognition-associated metabolites with three cognitive scores in a US-based cohort, in which data from the same metabolomic platform were available for 10 out of the 14 metabolites.

Furthermore, to ensure the relevance of our findings for AD, we also compared the cross-sectional metabolomic signature of cognition from the discovery cohort with the signature of incidence of AD prospectively in RSI-4. For this analysis, we included metabolomics data from 355 additional participants who were dementia-free at the time of metabolomics measurement but were diagnosed with AD during longitudinal clinical follow-ups, yielding a total of 1,229 RSI-4 participants available for Cox regression.

Characteristics of participants from the RS discovery and replication cohorts are summarized in Table 1."

Comment 4: For the first aim of evaluating brain MRI with distinct blood metabolites, I am wondering whether the authors have decided to use a hypothesis driven approach to evaluate each metabolite with the outcomes rather than using a data driven approach or machine learning methods (such as elastic net regressions) to identify metabolite signatures of the outcomes.

Response: The main goal of this first analysis was to establish a foundation for disentangling and revealing potential metabolic routes through which genetic risk, lifestyle factors, and the exposome may convey their influence on brain health and function during midlife (rather than to predict cognitive decline). To not miss any potentially relevant cognition-metabolite-determinant relationship (e.g., through regularization during training of a machine learning model), we first assessed associations of individual metabolites with cognitive performance and MRI markers irrespective of correlations among metabolites (e.g., between those linked to the same risk factor) and, in a second separate, orthogonal approach, linked each metabolite to its possible "source", i.e., to genetic and modifiable factors affecting the metabolite's blood levels. Also, we believe that the full set of univariate metabolite associations to any of the available markers of brain health (as well as any of the investigated determinants) as provided by our study, facilitates comparison and reuse of our results in future studies applying varying metabolomics approaches, which is less straight-forward in case of a concise signature of e.g., cognition resulting from a machine learning model.

Nonetheless, we fully agree that applying a machine learning approach as suggested by this reviewer certainly adds important information in the context of our study, in particular valuable estimates of how much variance in the cognition and MRI outcomes can be explained by the metabolome as a whole. We therefore utilized elastic-net regression for modelling general cognition and MRI variables using all metabolites, and included the overall variance explained (R^2) in each outcome in the revised manuscript. We also provide the sets of metabolites with non-zero beta estimates from the elastic-net models as multivariate signatures for their comparison with the results from the univariate models (**Supplementary Table 3**): Using elastic-net regression, we found that metabolites explained 10% of the variance in general cognition. The highest R^2 was observed for total brain volume, where 32.69% of the variance was explained. For hippocampal volume and white matter lesions, the explained variance was 17.5% and 1.98%, respectively. Among the top 20 contributors to general cognition were ergothioneine and uridine, both of which also appeared in the single-metabolite associations. For white matter lesions, S-adenosylhomocysteine (SAH) emerged as the top contributor, consistent with the univariate results. The details of the analyses and findings have been incorporated into the **Methods (lines 807-814)** and **Results (lines 164-168)** sections of the revised manuscript, respectively.

Comment 5. Have the authors considered for this first analysis using a more complete model including other variables such as smoking, hypertension or diabetes.

Response: In the original submission, we did not perform the association analysis using a regression model adjusted for smoking, hypertension, and diabetes, since we were specifically interested in metabolites that might mediate the effects of one or more of these AD risk factors on markers of brain health in midlife. While stepwise adjustment would certainly have been an alternative approach to identify such metabolites and their link to clinical or lifestyle factors, we saw two main advantages in the separated analyses, one for identifying metabolites associations with cognition/MRI markers (linear regression), and one for assessing the influence of clinical, lifestyle, microbial, and genetic factors (Gradient Boosting Decision Trees (GBDT)): (i) the results from the separate GBDT analysis provided for each metabolite can serve as a resource, enabling future look-ups of which and how much factors (genetic, microbial, clinical, medication, lifestyle) explain a metabolite's variance independent of the outcome of interest; (ii) potential non-linear relationships of microbial features in influencing a metabolite might be better captured by the GBDT approach.

Nonetheless, acknowledging the important further perspective provided by more complete models, we applied a third model, including the suggested additional covariates (smoking, hypertension, diabetes), as a sensitivity analysis. While all 14 (12) metabolites associated with cognition in the original model 1 (model 2) also showed evidence of association (P < 0.05) when we added smoking, hypertension, and diabetes as covariates, the associations did not reach *FDR* significance when correcting for testing 991 metabolites. In the association analyses of MRI markers using the fully adjusted model (original model + smoking, diabetes, and hypertension), all 22 originally associated metabolites were still associated with MRI phenotypes at P < 0.05. After correcting for 3 (phenotypes) x 991 (metabolites) tests, 7 of the 22 metabolites remained significant at *FDR* < 0.05. All results from these models are provided as supplementary information (general cognition: in the discovery cohort see **Supplementary Table 1: model 3** and in the replication cohort see **Supplementary Table 6: model 3**; MRI markers: see **Supplementary Table 2: model 2.**) Also, we incorporated a summary of the results described above in the revised manuscript (**Results, lines 126-129** and **153-156**).

Comment 6. The authors could clarify whether GWAS was conducted for each metabolite individually or in combination.

Response: We apologize for this omission in the original manuscript. We first performed GWASs for each metabolite separately using HASE (High-dimensional Association Study) software to prioritize variants with marginal significance of association (P <5 x10-8). In total, 415 independent SNPs were associated at P <5 x10^{-8} with at least one of the 991 metabolites after SNP clumping. For each metabolite, only those SNPs

showing association to the metabolite were included in the GBDT model to determine the percentage EV by genetics. We rephrased the description of the GWAS in the Methods section to clarify **(lines 905-907):**

"First, we performed a genome-wide association study (GWAS) for each of the 991 metabolites individually to prioritize variants with marginal significance of association with any metabolite (P < 5x10-8), using the HASE software (90). …"

Comment 7. Discussion. It would be good to highlight better the novelty of this work in the discussion as well as the clinical implications.

Response: We thank the reviewer for this valuable comment. The core novelty of our study, we see in the combination of two study types that mostly have been pursued "in isolation" in previous literature, namely a metabolome-wide association study of markers of brain health and function, on the one hand, and a comprehensive study of metabolite explained variance, on the other hand. Starting from a broad data-driven approach, we hypothesized that this combination would enable us to uncover scenarios where a cognition-associated metabolite potentially mediates an effect of a modifiable (e.g., lifestyle) factor on brain function. Using this combined approach as proposed in our study, we indeed revealed the potential role of ergothioneine as a mediator in the relationship between antacids and cognition; to the best of our knowledge, this finding is novel. Various previous studies matching the first study type investigated metabolite association to cognition and/or MRI markers but did not explore the potential sources of these metabolites or their role as mediators of various exposomal risk factors (references 5-15 in the manuscript cite examples of such studies). Also, the majority of these brain health related studies focused on elderly cohorts while studies in midlife populations are still generally scarcer for this phenotype.

Following the reviewer's advice, we have included a short paragraph highlighting this key novelty of our work in the Discussion of the revised manuscript (**lines 436-448**):

"In this study, we provide an integrated evaluation of how circulating metabolites relate to brain health in midlife and how these metabolites are shaped by modifiable exposures across the exposome. By combining a metabolome-wide analysis of cognition and MRI markers with a systematic assessment of genetic, microbial, lifestyle, clinical, and medication determinants of metabolite levels in the population-based Rotterdam Study, we showed that this novel integrative approach can delineate potential metabolic routes through which modifiable factors may influence early brain alterations relevant to AD. Notably, our findings highlight ergothioneine as a metabolite strongly linked to cognition and incident AD risk, and revealed for the first time that antacid medication may be a relevant determinant of its circulating levels. While not proving causality, our mediation analysis in the available cross-sectional dataset suggests that reduced ergothioneine may partly convey the adverse cognitive association observed

with antacids, offering a novel and potentially actionable route through which medication use could affect brain health, if confirmable in longitudinal studies."

In relation to the second study type focusing on metabolite explained variance, the main advancement by our study over previous ones mainly lies in the addition of EV estimates for the influence of common drugs. Most existing metabolite EV studies were based on younger, very healthy individuals with limited medication intake, which was mentioned as an explicit limitation in one of these large previous studies. Given the added value of medication-related EV derived in our study with respect to existing literature, we provide the results for all 991 analyzed metabolites for their use in future studies. In the revised version of our manuscript, we more explicitly highlight how our study advances existing EV studies in the Discussion (**lines 573-583)**, thereby also addressing Comment 5 by Reviewer #2:

"One of the main strengths of our study is its dual approach, integrating the identification (and replication) of metabolites linked to midlife brain health with a systematic exploration of the extent to which metabolites are influenced by genetic and potentially modifiable exposomal factors. While the incorporation of EV analysis in the study of cognition-related metabolite signatures is novel, various previous studies thoroughly investigated explained variance in metabolomics in isolation, particularly exploring influences of genetic, gut microbial, and dietary features (25-28). Our study extends this previous work by adding variance explained by medication use for 991 metabolites, using intake data on 31 common medications in our middle-aged cohort. Notably, in Bar et al. (25), the limited drug intake by the relatively young and healthy study participants used for EV estimation, was explicitly discussed by the authors as a limitation resulting in probably underestimated medication-related EVs."

Although our results suggest a potential negative effect of antacid/proton pump inhibitor use on cognition during midlife, mediated by lower levels of the neuroprotective metabolite ergothioneine, we believe that further confirmatory studies are required before any implications for clinical practice can be considered. In particular, longitudinal clinical studies (e.g., following medication-naïve individuals after their first proton pump inhibitor prescription) and mechanistic investigations are needed to clarify causality and underlying pathways. In response to the reviewer's suggestion, we have added the following paragraph, mentioning the potential clinical relevance of these findings while using careful language to avoid overinterpretation (**lines 621-626**):

"Consequently, the mediating role of ergothioneine warrants confirmation in longitudinal cohorts, where the temporal order of antacid (e.g., protein pump inhibitor) intake and the decline of ergothioneine levels and cognitive performance can be clearly delineated. Also, further mechanistic work is needed before any conclusions on potential clinical implications, such as reconsideration of the recommendations for the prescription of proton pump inhibitors or other antacids, can be drawn."

Reviewer #2

Ahmad et al. examined associations between 991 plasma metabolites, cognition, and MRI-derived neuroimaging markers in two Rotterdam Study cohorts. They quantified variance in metabolites attributable to genetics, gut microbiota, lifestyle, clinical factors, and medications. Fourteen metabolites were linked to cognition, with nine replicating in an older cohort and aligning directionally with incident AD. Ergothioneine, identified as neuroprotective, showed reduced levels with antacid use and partially mediated the antacid-cognition relationship.

Major comments

Comment 1. The study lacks a clear hypothesis (e.g., biomarker discovery, mechanistic insight, or clinical prediction). Clarify aims and align results presentation accordingly (e.g., include independent replications, mechanistic experiments, or predictive performance). The current replication population is from part of the discovery population, and therefore, the validity of replication is somehow undermined.

Response: We thank the reviewer for this important comment and apologize for the lack of clarity regarding the study's hypothesis. Our primary aim was to identify circulating metabolites associated with cognitive function in midlife as potential biomarkers linking genetic and modifiable metabolic risk factors to brain health and AD risk. As such, our study can be considered a biomarker discovery study; but instead of focusing on finding biomarkers for the prediction of cognitive decline or early diagnosis of AD, we were after metabolic biomarkers that are linked to specific (modifiable) risk factors of dementia and might play a role as potential mediators of risk during midlife.

Following the reviewer's suggestions, we have added text to clarify these aims and the underlying hypothesis in the Introduction (**lines 45-66**) and included a second replication analysis using data from a US cohort (Results: **lines 202-223**; Methods: **lines 838-859**). Additionally, we would like to emphasize that the participants of the original, first replication cohort (RSI-4) were not part of the discovery cohort (RSIII-2) but were recruited in independent surveys (recruitment RSI: 1989-1992; RSIII: 2006-2008; we rephrased the study description in the Methods section to clarify (**lines 649-651**). Nonetheless, we fully agree with this reviewer that using a cohort from the same relatively small geographic region for replication puts clear limits regarding the proof of the generalizability of findings. In the newly added replication analysis in 512 participants of a US-based study, we were able to replicate seven out of the ten cognition-associated metabolites, for which data was available. Together with the first replication in RSI-4, all cognition-associated metabolites replicated in at least one of the two replication cohorts. The respective passages in the manuscript now read:

Clarification of aims/hypothesis - Introduction, lines 45-66:

"As the gut microbiome and many factors of the exposome are modifiable in nature, their management provides an opportunity to stabilize metabolism and counteract disease-related metabolic alterations. However, to define targeted interventions and

prevention strategies, a profound understanding is required of (i) the metabolites associated with early alterations in brain structure and cognitive function related to AD, and (ii) the extent to which these metabolite levels are influenced (and thus potentially modifiable) by the gut microbiome and the exposome in pre-symptomatic participants. Despite increasing knowledge on metabolites associated with cognition, MRI markers, and AD diagnosis in elderly, the implications of metabolites for brain health in midlife, their specific interplay with risk factors and potential role as mediators of risk are not well understood.

In this study, we aimed at filling this research gap by systematically combining the identification of early metabolic markers of brain health with the analysis of their potential determinants, including various metabolism- and AD-related risk factors. We hypothesize that this integrated analysis can pinpoint metabolites that may partially convey the effects of exposomal and lifestyle factors on brain function with potential consequences for prevention. Specifically, we first investigated the cross-sectional association of circulating metabolites with general cognition and MRI markers in midlife within a dementia-free sample of the population-based Rotterdam Study (RS) to identify potential biomarkers of brain health. The resulting cognition-associated metabolites were replicated cross-sectionally in two independent cohorts of older individuals. To ensure the relevance of these metabolites for AD, they were further tested for their relationship with incident AD diagnosis during follow-up in one of the replication cohorts in a prospective design. In a second step, we evaluated the contribution of genetic variation, gut microbiome, various lifestyle factors, medication use, and clinical features to define levels of identified metabolites in circulation in the discovery cohort and investigated the interplay of these factors in detail for selected examples, exploring their potential as targets for intervention and prevention. …”

Additional replication - Results, lines 202-223:

”We replicated the findings obtained from the cross-sectional association of metabolites with general cognition in this study (RSIII-2 cohort) in 847 participants from the RSI-4 cohort, which were recruited independently from the same region in The Netherlands 15 years before RSIII recruitment. Among the 14 metabolites associated with general cognition in RSIII-2 (FDR < 0.05), nine metabolites showed significant association (P < 0.05) in the cross-sectional replication analysis in RSI-4 with concordant direction of association (Table 2).

For further replication, we used data from seven Alzheimer’s Disease Research Centers (ADRC) across the United States collected for 512 subjects as part of the Alzheimer Gut Microbiome Project (AGMP). We tested the cross-sectional associations of three cognitive scores (CRAFTDRE - Craft story delayed recall, UDSBENTD - Uniform Data Set Benson figure delayed recall, NACCMOCA - education adjusted total Montreal Cognitive Assessment score) with ten out of the 14 cognition-associated metabolites, for which plasma levels were available (measured using the same metabolomics platform as used in the discovery cohort). Seven of these ten metabolites showed significant association

(P < 0.05) in at least one of the three cognitive scores (Supplementary Table 4), including ergothioneine (CRAFTDRE: 0.221, P = 4.50x10^{-3}; NACCMOCA: 0.019, P = 2.36x10^{-2}), 2-naphthol sulfate (CRAFTDRE: -0.060, P = <1.00x10^{-4}; UDSBENTD: -0.049, P = 2.90x10^{-3}; NACCMOCA: -0.055, P = 4.00x10^{-4}), and uridine (CRAFTDRE: 0.009, P = <2.37x10^{-2}; UDSBENTD: 0.013, P = 2.60x10^{-2}; NACCMOCA: 0.013, P = 1.00x10^{-3}), and the four metabolites with yet unknown chemical structure. Thus, all 14 metabolites from the discovery analysis showed significant association (P < 0.05) in at least one of the four tests (general cognition in RSI-4 cohort; CRAFTDRE, UDSBENTD, NACCMOCA in AGMP cohort) performed for replication."

Comment 2. Assess whether cognition-associated metabolites predict longitudinal cognitive decline within the Rotterdam cohort to strengthen biomarker relevance.

Response: While the main focus of our study was not on prediction of cognitive decline, we fully agree that linking the cognition-associated metabolites to future cognitive decline is important to judge their relevance for the development of AD. In the original version of the manuscript, we therefore assessed the metabolite signature of incident AD in the older participants of RSI-4 longitudinally (Cox proportional hazard analysis) and compared it to the cross-sectional signature of cognition as revealed in RSIII-2. The observed good concordance of these signatures did support the relevance of our findings for AD. Nonetheless, testing and confirming the potential of the cognition-associated metabolites to predict cognitive decline in RSIII participants as suggested by the reviewer could certainly strengthen the confidence in their link to dementia further. Unfortunately, phenotypic data from only one follow-up visit (RSIII-3) have become available for 510 participants of RSIII-2 since the visit used for the cross-sectional analyses. In longitudinal analyses, using linear mixed-effect models of general cognition in this data set (**lines 795-806**), none of the 14 metabolites showed significant interaction with follow-up time, probably due to the limited sample size and follow-up time.

To address the reviewer's comment and reconfirm the importance of the identified metabolites for AD beyond the described support through the similarity of metabolite signatures in RSI-4, we added further citations to recent metabolite studies that focused on predicting cognitive decline in population-based settings or faster progression to AD in patient cohorts: Especially for ergothioneine, evidence for its relevance is continuously growing: For example, very recently, Zhao et al. [1] reported that lower ergothioneine levels in blood associated with longitudinal decline in cognitive function in a Chinese cohort of non-demented participants. Furthermore, low levels of ergothioneine have been shown to predict progressive mild cognitive impairment in AD patients [2]. Also, low uridine levels were associated with disease progression in AD [3]. We included these additional references in the Discussion of the revised manuscript. Moreover, we extended the paragraph on the limitations of our study to acknowledge

the need for further longitudinal data to gain deeper insights in the role of the identified cognition-associated metabolites for healthy brain aging (**lines 606-611):**

"A further limitation is the so far limited follow-up of the participants of our study with longitudinal cognitive assessments and metabolomics data, which may provide additional insights into the role of metabolites in cognitive decline over time. In the available data on general cognition from 510 RSIII-2 participants, the metabolites cross-sectionally associated with cognition did not show significant association with the change in cognition over time. …"

Comment 3. Please report the distribution of blood collection-to-MRI scan intervals, and test whether the significant metabolite-MRI marker associations persist after excluding individuals with long intervals (e.g., >1 year).

Response: We thank the reviewer for this valuable comment. In our MRI sample (N = 925), the mean interval between blood collection and MRI was 0.86 years (SD = 1.54), with a median of 0.18 years (IQR = 0.97) and a range of 0 to 7.97 years (**Table R1**). We have incorporated this information in the revised manuscript **(lines 773-775)** and performed the suggested sensitivity analysis excluding participants with more than a one-year interval between blood collection and MRI.

Table R1: Distribution of collection-to-MRI scan interval years

N	Mean	SD	Median	IQR	Min	Max
925	0.864	1.536	0.178	0.972	0.000	7.978

Testing all 991 metabolites for their association with total brain volume and total white matter lesions in the remaining 688 participants with intervals <1 year, 15 of the 22 metabolites that were associated in the full sample remained significantly (*FDR* < 0.05) associated with the MRI markers (**Supplementary Table 2**); all 22 metabolites were still associated at *P* < 0.05 (**Table R2**). We have included this analysis in the revised manuscript (Results: **lines 156-163**; Methods: **lines 792-794**), **Supplementary Table 2**).

Table R2: Sensitivity analysis results for MRI-associated metabolites

MRI Phenotype	BIOCHEMICAL_NAME	Beta	Se	p	n
Total_brain_volume(ml)	1,3,7-trimethylurate	-0.065	0.013	6.67E-07	688
Total_brain_volume(ml)	theophylline	-0.055	0.013	1.99E-05	688
Total_brain_volume(ml)	caffeine	-0.053	0.013	4.72E-05	688
Tota_brain_volume(ml)	1,3-dimethylurate	-0.052	0.013	7.00E-05	688
Total_wml	S-adenosylhomocysteine (SAH)	0.148	0.037	6.57E-05	688
Total_brain_volume(ml)	argininate	0.049	0.013	1.59E-04	688
Total_brain_volume(ml)	(S)-3-hydroxybutyrylcarnitine	-0.050	0.013	1.43E-04	688
Total_brain_volume(ml)	glutamine conjugate of C6H10O2 (2)	-0.049	0.013	1.64E-04	688
Total_brain_volume(ml)	X - 26107	-0.051	0.013	1.56E-04	688
Total_brain_volume(ml)	glutamine conjugate of C6H10O2 (1)	-0.048	0.013	2.03E-04	688
Total_brain_volume(ml)	paraxanthine	-0.048	0.013	2.30E-04	688
Total_brain_volume(ml)	3-hydroxyhexanoylcarnitine (1)	-0.044	0.013	7.31E-04	688
Total_brain_volume(ml)	3-hydroxyoleoylcarnitine	-0.044	0.013	8.24E-04	688

Total_brain_volume(ml)	sphingomyelin (d18:2/24:2)	0.046	0.014	1.14E-03	688
Total_brain_volume(ml)	cyclo(leu-pro)	-0.042	0.013	1.64E-03	688
Total_brain_volume(ml)	glycerophosphorylcholine (GPC)	0.041	0.013	1.84E-03	688
Total_brain_volume(ml)	N-lactoyltyrosine	-0.042	0.014	1.95E-03	688
Total_brain_volume(ml)	sphingomyelin (d18:1/20:1, d18:2/20:0)	0.042	0.014	2.28E-03	688
Total_brain_volume(ml)	3-hydroxysebacate	-0.038	0.013	3.83E-03	688
Total_brain_volume(ml)	sphingomyelin (d18:2/18:1)	0.038	0.014	5.21E-03	688
Total_brain_volume(ml)	X - 11787	0.032	0.013	1.22E-02	688
Total_brain_volume(ml)	6-bromotryptophan	0.026	0.013	3.94E-02	688

Comment 4. Mediation assumes a temporal sequence between the exposure, mediator, and outcome, which the manuscript does not seem to establish. A clear study timeline should be provided to justify the mediation analysis. Furthermore, the mediated association (antacids, ergothioneine, and cognition) is novel and interesting, however, the underlying mechanism is unclear based on the current literature. I would tone down the relevant discussion.

Response: We acknowledge that our mediation analysis assumes a temporal sequence between the exposure, mediators, and outcome, which cannot be fully established given the cross-sectional nature of our data. Information on antacid medication use was extracted from patient medical records; blood samples (for ergothioneine assessment) and cognitive measurements were collected during the Rotterdam Study III second follow-up visit. Thus, exposure, mediator, and outcome were assessed at the same follow-up. As advised by this reviewer, we have toned down the discussion of our mediation finding in the revised discussion, pointing out that it should be interpreted with caution and that confirmation in longitudinal cohorts, where the temporal order can be clearly delineated, is warranted.

Discussion, lines 499-506:

"… Our mediation analysis results suggest the reduction of ergothioneine as one possible mechanism explaining harmful effects of this medication on neurodegenerative diseases. However, this finding should be interpreted with caution as the temporal sequence of events was assumed for mediation analysis but cannot be ascertained in our cross-sectional dataset of middle-aged participants where exposure (antacid), mediator (ergothioneine), and outcome (cognition) were assessed at the same visit. Also, it remains unclear whether these medications affect the absorption of ergothioneine from diet as has been shown for vitamin B12 (42) or whether they influence ergothioneine levels by other means."

Discussion, lines 520-524:

"In summary, these pieces of evidence point towards a possible role of ergothioneine for maintaining cognitive function and underscore the value of further research into intervention and prevention schemes targeting the management of ergothioneine. More research is also needed to confirm ergothioneine as a potential mediator of the still controversial relationship between PPIs and dementia risk."

Discussion (limitations), lines 617-626:

"… Finally, in mediation analyses, we assumed a temporal sequence of exposure, mediator, and outcome. However, due to the cross-sectional nature of our analysis, our mediation findings should not be interpreted as evidence of causation. In general, we cannot infer causality through any of the analyses performed in this study. Consequently, the mediating role of ergothioneine warrants confirmation in longitudinal cohorts, where the temporal order of antacid (e.g., protein pump inhibitor) intake and the decline of ergothioneine levels and cognitive performance can be clearly delineated. Also, further mechanistic work is needed before any conclusions on potential clinical implications, such as reconsideration of the recommendations for the prescription of proton pump inhibitors or other antacids, can be drawn."

While the mediating role of ergothioneine needs further confirmation, please note that we were able to at least replicate the association of proton pump inhibitor intake with lower ergothioneine blood levels in the AGMP replication cohort. To the best of our knowledge, our study is the first to report on this link between antacid medication and ergothioneine. Considering the novelty of this finding, we included this replication result in the revised manuscript (Results: **lines 408-410**, Discussion: **lines 492-493**).

Comment 5. Clarify if sex-stratified analyses are exploratory and address statistical power for detecting sex-specific effects.

Response: The reviewer is correct that sex-stratified analyses were exploratory. We apologize for not having stated this more clearly in the original manuscript. Please note, that, in response to a comment by Reviewer #3, we have updated the stratified analysis and its description in the revised manuscript, following the reviewer's suggestions (please see our response to Comment 1 of Reviewer #3 for more details).

To address this comment (Comment 5), we estimated the statistical power for detecting sex-specific effects based on the sex-interaction effect observed for the association of cognition with **N-acetyl-aspartyl-glutamate (NAAG)**, the metabolite showing the strongest difference between the sexes in the updated analysis. While a ~ 80% power of detecting the observed effect size was estimated at a nominal significance threshold of $\alpha = 0.05$, the power drops down to ~ 10% when considering Bonferroni adjustment for testing 991 metabolites (Methods: **lines 823-828**). In the revised manuscript, we have added a note, clearly indicating this lack of power as a limitation (**lines 604-606**):

"Second, our study did not have sufficient power (~10%, see Methods) to reliably identify sex-specific metabolite association with cognition or MRI markers considering multiple testing correction."

Comment 6. I have a concern regarding the novelty of the explained variation analysis, considering the 2022 Nature Medicine article, "Influence of the microbiome, diet and genetics on inter-individual variation in the human plasma metabolome", which explored similar aspects

of exposome and microbiome interactions with the metabolome. The authors should clearly articulate how their findings advance beyond previous work, contrast their variance estimates with those reported in the Nat Med article, and discuss discrepancies.

Response: We certainly acknowledge that previous studies, including the study by Chen et al. [4] (ref. 26 in the original manuscript), have reported explained variance analyses in metabolomics, particularly for genetic and gut microbial features, before. We apologize that this was not explicitly highlighted in the original version of our manuscript.

In our study, we employed explained variance analysis on further modifiable factors (including medication use) and systematically integrated these new findings with the role of metabolites in cognition, MRI measures, and Alzheimer's disease outcome. Specifically, we incorporated explained variance of blood metabolites based on 31 commonly used medications as relevant features of the exposome (rather than covariates) and investigated their relationship to midlife cognition, which has not been addressed in previous publications. We thereby found evidence for a link between antacid medication and lower levels of ergothioneine, which has not been reported on before to the best of our knowledge. Moreover, Chen et al. and our study differ in metabolomics profiling strategies and therefore cover complementary sets of metabolites. Based on mapping HMDB IDs, only 133 metabolites overlapped between our Metabolon dataset and the published study (out of 1,183 (Chen et al.) and 991 (our study)). Thus, our study investigates a different set of metabolic features, many of which were not included in the 2022 Nature Medicine article (e.g., ergothioneine).

Taken together, our work advances beyond this prior work by (i) integrating metabolite explained variance analysis with a metabolome-wide association study on brain health in midlife (-> *integrative analysis with cognition*); (ii) expanding metabolite explained analysis to further variables (especially medication use) and using a complementary metabolite panel (-> *expanded/complementary analysis*); (iii) reporting first evidence for ergothioneine as potential mediator of antacids' effect on cognition (-> *novel results*).

Following this reviewer's advice, we expanded the discussion of how our work advances beyond previous studies on metabolite explained variance (Discussion: **lines 573-596**). We also included a detailed comparison of EV results from Chen et al. and our study for the two feature classes that overlap between the studies, namely genetics and gut microbiome (**Supplementary Material**).

Discussion, 572-596:

"One of the main strengths of our study is its dual approach, integrating the identification (and replication) of metabolites linked to midlife brain health with a systematic exploration of the extent to which metabolites are influenced by genetic and potentially modifiable exposomal factors. While the incorporation of EV analysis in the study of cognition-related metabolite signatures is novel, various previous studies thoroughly

investigated explained variance in metabolomics in isolation, particularly exploring influences of genetic, gut microbial, and dietary features (25-28). Our study extends this previous work by adding variance explained by medication use for 991 metabolites, using intake data on 31 common medications in our middle-aged cohort. Notably, in Bar et al. (25), the limited drug intake by the relatively young and healthy study participants used for EV estimation, was explicitly discussed by the authors as a limitation resulting in probably underestimated medication-related EVs. However, the EV analysis in our study also comes with various limitations. First, dietary information was not available for the time of blood collection. As diet is known to be an important lifestyle factor defining the levels of many metabolites, this lack of data clearly limits the insights about the impact of the exposome. Also, the impact of physical activity could not be investigated in this study. Second, the estimation of explained variance strongly depends on the features used as input. For example, applying a less strict threshold for the inclusion of genetic variants will result in overall higher estimates for the genetic contribution for all metabolites. With the chosen approach the EVs for genetics were similar to those previously reported by Bar et al. (25). Also, metabolites driven by gut-microbiota showed similar EV percentages when compared to two previous studies (25, 26), underscoring the use of EV estimates across studies in this case (Supplementary Material). Differences in the EV of metabolites by lifestyle factors and clinical variables with these earlier studies may be attributable to the difference in the inclusion of various features in these types of predictors."

Supplementary Material:

"For genetic and gut microbiome determinants, we compared the estimates for the explained variance of metabolite levels from our study with the ones reported in Chen et al. (1) for the 133 metabolites measured in both studies (Supplementary Table 8). In Chen et al. and our study, 11 and 17 metabolites showed genetic contribution (EV > 0) in this set, respectively, with variance being explained through genetics for five metabolites in both studies: N(6)-methyllysine (our study: 61.6 % vs. Chen et al.: 15.8 %), N6,N6-dimethyllysine (41.4 % vs. 9.2 %), N-acetylornithine (23.1 % vs. 14.5 %), cis-4-decenoate (10:1n6) (2.8 % vs. 3.2 %), and 1-arachidonoyl-GPC (20:4n6) (1.9 % vs. 4.5 %). Notably, while the overlap seems moderate, the metabolites with EV>0 for genetics in only one of the studies have been identified (and replicated) as significant hits in previous studies on metabolite quantitative trait loci (mQTL) (e.g., (2)). For microbial features, the explained variance was more consistent across the two studies, with, for example, indolepropionate (our study: = 15.8 % vs. Chen et al.: 12.2 %), p-cresol sulfate (31.9 % vs. 23.8 %), hippurate (16.1 % vs. 13.4 %), and phenol sulfate (11.0 % vs. 11.5 %) showing similar levels of variance explained."

Please note that we have combined these new analyses with the comparison of EV results between our study and those from Bar et al. that was part of the discussion in the original version of our manuscript.

Minor comments

Comment 7. Page 7: Specify the exact annotation for "glutamine conjugates of C6H10O2 (2)."

> **Response:** For the analytical signals labeled as glutamine conjugates of C6H10O2 (1) and (2) it has not been possible to specify the underlying chemical structure of the molecules beyond their characteristic losses of a glutamine moiety. Therefore, the two metabolites were described as “partially characterized molecules” by Metabolon, corresponding to metabolite identification level 3 (i.e., partial chemical annotation according to plausibility and comparison with public spectral libraries) according to the levels of metabolite identification as defined by the Metabolomics Standardization Initiative (MSI) [5]. To address the reviewer’s comment, we mention that the analytes named “glutamine conjugates of C6H10O2” are partially characterized molecules when reporting related results in the revised manuscript. Moreover, we added a column to **Supplementary Table 1**, providing the MSI metabolite identification level for each metabolite.

Comment 8. Nomenclature: Ensure consistency (e.g., "3-methylcatechol sulfate" vs. "3-methyl-catechol sulfate (2)").

> **Response:** We went through all text, figures, and tables of the manuscript to ensure consistent nomenclature of metabolites.

Comment 9. Figure 2: Clarify labeling criteria for significant metabolites; resolve discrepancy between labeled (10) and described (9) replicated metabolites.

> **Response:** We apologize for the discrepancy. We thought to label only those metabolites that were significantly associated with general cognition in RSIII-2 and replicated in the RSI-4 cohort. Uridine was labeled by mistake as it did not replicate in RSI-4 (though it did in the newly added replication in the US cohort). We explicitly mention the criterion for labelling in the legend of Figure 2 now. We additionally labeled the two metabolites most strongly associated with incidence of AD as those are mentioned in the text.

Comment 10. Figure S3: Use bounding boxes/shading to group metabolite clusters in the correlation matrix for clarity.

> **Response:** Thank you for this valuable suggestion. We amended the Figure accordingly.

Comment 11. Figure S7: Add the missing column header in the heatmap.

> **Response:** We apologize for this mistake and corrected it in the revised version of Supplementary Figure 7.

Reviewer #3

This work present significant originality and significance a it integrates blood metabolomics (991 metabolites) with neuroimaging and cognition data, which is still relatively novel, especially at this scale and depth. It not only identifies associations between metabolites and brain measures but also assesses the sources of variance in metabolite levels, including:
Genetics, Lifestyle, Comorbidities, Medication and Gut microbiota.
Overall, this is a very well written manuscript, with appropriate use of statistics.
In this study, 991 frequent metabolites in 1,068 participants were characterized through the untargeted commercial platform Metabolon in the Rotterdam study III and associated with cognition and brain imaging (pre-AD diagnosis). For cognition, the findings were replicated in 874 participants of the older RSI-4 cohort and in a later follow up with patients with incident AD (355). This study constitutes a large sample size for this type of analysis with replication which re-enforced the findings.

> **Response:** We greatly value this favorable evaluation of our work and are grateful for the valuable advice regarding the sex-stratified analysis.

Minor comment 1: Results from the sex stratified analyses should be summarized differently. The first step would be to run models with an interaction term between metabolite and sex and stratify in the case of a p-value under 0.05 for example. Then, the authors should report effect sizes in both males and females and not just p-values of individual models.

> **Response:** Thank you for this helpful comment. We fully agree that the suggested interaction analysis is needed to identify actual sex-specific associations. Following the reviewer's advice, we applied the following model for general cognition and MRI phenotype outcomes in the RSIII-2 discovery cohort and for general cognition also in the RSI-4 replication cohort.
>
> **Model: Outcome ~ Metabolite * Sex + Age at blood collection + BMI + Lipid-lowering medication (for MRI additional collection-to-MRI time and intra-cranial volume)**
>
> For general cognition and MRI markers, 22 and 61 metabolites showed evidence of heterogeneity between males and females (*P* value of interaction < 0.05) (**Supplementary Table 5**), respectively. In addition to the detailed results from the interaction analysis, we also provide the effect size estimates, standard error, and p-value from the sex-stratified analysis in **Supplementary Table 5**. In the revised manuscript, we included a short subsection, describing the results from these analyses (**lines 242-263**):
>
> *"On Sex-stratified analysis suggests association of bile acids with cognition and MRI markers in females*
>
> *To explore potential sex differences in metabolite associations with general cognition and MRI markers, we tested for significant sex-metabolite interactions when modelling these markers of brain health. We found suggestive evidence of interaction with sex (P interaction < 0.05) for 22 and 61 metabolites, in their association with general*

cognition, and MRI markers, respectively (Supplementary Table 5), and further examined these metabolites in sex-stratified analyses (females = 491, males = 407). Lower blood levels of five metabolites were significantly (FDR < 0.05) associated with better general cognition only in female participants: 7-alpha-hydroxy-3-oxo-4-cholestenoate (7-Hoca) (adjusted mean difference = -0.086, P = $9.83x10^{-3}$), taurocholate (-0.093, P = $5.81x10^{-3}$), glycocholate (-0.096, P = $4.15x10^{-3}$), methyl indole-3-acetate (-0.092, P = $5.84x10^{-3}$), and 13-HODE + 9-HODE (-0.117, P = $5.41x10^{-3}$) (FDR < 0.05). In contrast, higher levels of N-acetyl-aspartyl-glutamate (NAAG) (0.120, P = $5.15x10^{-3}$) were associated with better cognition only in male participants. Ten metabolites showed significant associations with total brain volume in females, six metabolites with total hippocampal volume, and five metabolites with white matter lesions, also restricted to females. Among the six metabolites associated with hippocampal volume, lower levels of five conjugated bile acids (glycocholate, taurocholate, glycochenodeoxycholate, taurochenodeoxycholate, and taurodeoxycholate) were associated to larger hippocampal volume in females. In addition, six metabolites showed male-specific associations with MRI phenotypes (Supplementary Table 5). However, none of these sex-stratified findings replicated in the RSI-4 cohort (Supplementary Table 5)."

Unfortunately, none of the interaction results in RSIII-2 replicated in RSI-4. According to a power calculation, which was suggested by Reviewer #2, this might be due to the limited statistical power to detect sex differences in our study. We discuss the related limitations in the revised manuscript (see our response to Comment 5 by Reviewer #2).

References

1. Zhao, D., et al., *Associations of plasma ergothioneine levels with cognitive function change in non-demented older Chinese adults: A community-based longitudinal study.* medRxiv, 2025: p. 2025.07. 16.25331363.
2. Oka, T., et al., *Multiomics analysis to explore blood metabolite biomarkers in an Alzheimer's Disease Neuroimaging Initiative cohort.* Sci Rep, 2024. **14**(1): p. 6797.
3. de Leeuw, F.A., et al., *LDL cholesterol and uridine levels in blood are potential nutritional biomarkers for clinical progression in Alzheimer's disease: The NUDAD project.* Alzheimers Dement (Amst), 2020. **12**(1): p. e12120.
4. Chen, L., et al., *Influence of the microbiome, diet and genetics on inter-individual variation in the human plasma metabolome.* Nat Med, 2022. **28**(11): p. 2333-2343.
5. Sumner, L.W., et al., *Proposed minimum reporting standards for chemical analysis Chemical Analysis Working Group (CAWG) Metabolomics Standards Initiative (MSI).* Metabolomics, 2007. **3**(3): p. 211-221.
